# Metabolomics of *Escherichia coli* for Disclosing Novel Metabolic Engineering Strategies for Enhancing Hydrogen and Ethanol Production

**DOI:** 10.3390/ijms241411619

**Published:** 2023-07-18

**Authors:** Antonio Valle, Maria Elena de la Calle, Howbeer Muhamadali, Katherine A. Hollywood, Yun Xu, Jonathan R. Lloyd, Royston Goodacre, Domingo Cantero, Jorge Bolivar

**Affiliations:** 1 Department of Biomedicine, Biotechnology and Public Health-Biochemistry and Molecular Biology, Campus Universitario de Puerto Real, University of Cadiz, 11510 Puerto Real, Spain; elena.decalle@gm.uca.es; 2 Institute of Viticulture and Agri-Food Research (IVAGRO)—International Campus of Excellence (ceiA3), University of Cadiz, 11510 Puerto Real, Spain; domingo.cantero@uca.es; 3 Department of Chemical Engineering and Food Technology, Campus Universitario de Puerto Real, University of Cadiz, 11510 Puerto Real, Spain; 4 Centre for Metabolomics Research, Department of Biochemistry, Cell and Systems Biology, Institute of Systems, Molecular and Integrative Biology, University of Liverpool, BioSciences Building, Crown Street, Liverpool L69 7ZB, UK; howbeer.muhamad-ali@liverpool.ac.uk (H.M.); yun.xu@liverpool.ac.uk (Y.X.); roy.goodacre@liverpool.ac.uk (R.G.); 5 Manchester Centre for Synthetic Biology of Fine and Speciality Chemicals (SYNBIOCHEM), Manchester Institute of Biotechnology, The University of Manchester, Manchester M1 7DN, UK; katherine.hollywood@manchester.ac.uk; 6 Department of Chemistry, Faculty of Science and Engineering, Manchester Institute of Biotechnology, The University of Manchester, Manchester M1 7DN, UK; 7 Williamson Research Centre, School of Earth & Environmental Sciences, University of Manchester, Manchester M13 9PL, UK; jon.lloyd@manchester.ac.uk; 8 Institute of Biomolecules (INBIO), University of Cadiz, 11510 Puerto Real, Spain

**Keywords:** hydrogen, ethanol, *Escherichia coli*, metabolomics, GC-MS, FT-IR

## Abstract

The biological production of hydrogen is an appealing approach to mitigating the environmental problems caused by the diminishing supply of fossil fuels and the need for greener energy. *Escherichia coli* is one of the best-characterized microorganisms capable of consuming glycerol—a waste product of the biodiesel industry—and producing H_2_ and ethanol. However, the natural capacity of *E. coli* to generate these compounds is insufficient for commercial or industrial purposes. Metabolic engineering allows for the rewiring of the carbon source towards H_2_ production, although the strategies for achieving this aim are difficult to foresee. In this work, we use metabolomics platforms through GC-MS and FT-IR techniques to detect metabolic bottlenecks in the engineered Δ*ldh*Δ*gnd*Δ*frdBC*::kan (M4) and Δ*ldh*Δ*gnd*Δ*frdBC*Δ*tdcE*::kan (M5) *E. coli* strains, previously reported as improved H_2_ and ethanol producers. In the M5 strain, increased intracellular citrate and malate were detected by GC-MS. These metabolites can be redirected towards acetyl-CoA and formate by the overexpression of the citrate lyase (CIT) enzyme and by co-overexpressing the anaplerotic human phosphoenol pyruvate carboxykinase (hPEPCK) or malic (MaeA) enzymes using inducible promoter vectors. These strategies enhanced specific H_2_ production by up to 1.25- and 1.49-fold, respectively, compared to the reference strains. Other parameters, such as ethanol and H_2_ yields, were also enhanced. However, these vectors may provoke metabolic burden in anaerobic conditions. Therefore, alternative strategies for a tighter control of protein expression should be addressed in order to avoid undesirable effects in the metabolic network.

## 1. Introduction

The development and application of metabolomics in the last two decades have improved our understanding of different biological processes [1,2,3]. The interest in establishing individual metabolic patterns dates back to the 1950s [4], but it was the implementation of analytical platforms such as Gas or Liquid Chromatography–Mass Spectrometry (GC–MS or LC-MS) and nuclear magnetic resonance (NMR) spectroscopy that boosted the application of metabolomics in different fields [5]. This approach is of extraordinary interest in metabolic engineering since one of the main goals of this research area is to make the most of existing metabolic pathways in order to divert resources toward compounds of industrial relevance. The production of a molecule depends on the availability of the carbon source, the energy, and cofactors as well as the existence of competitive pathways [6]. Therefore, the identification of metabolic bottlenecks will help to design novel and improved engineered strains [7].

**Figure 1:**
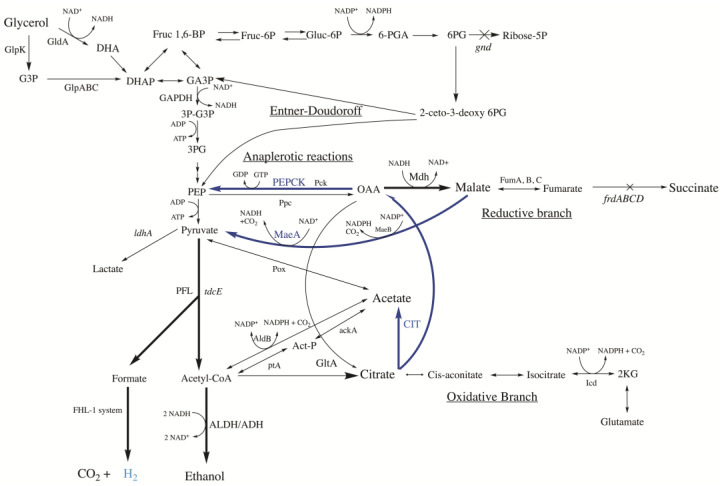
Diagram of the metabolic pathways of glycerol assimilation, TCA oxidative and reductive branches, anaplerotic reactions, and the fermentation of pyruvate into H_2_ and ethanol. The arrows show the reaction directions in accordance with the direction of enzyme catalysis that are theoretically possible under our experimental conditions. In the reductive branch, the enzymes involved in succinate reduction are: malate dehydrogenase (Mdh), fumarase isoenzymes (FumA, B, C), and fumarate reductase complex (FrdABCD). Several enzymes are involved in the anaplerotic reactions: the phosphoenol pyruvate (PEP) carboxylase (Ppc), the PEP carboxykinase (Pck), the NAD^+^-dependent malic enzyme (MaeA), and the NADP^+^-dependent malic enzyme (MaeB). In the oxidative branch, the remarked enzymes in this work are citrate synthase (GltA) and isocitrate dehydrogenase (Icd). Other enzymes and intermediates involved in these pathways are: AckA: acetate kinase, AldB: aldehyde dehydrogenase, ALDH/ADH: acetaldehyde/alcohol dehydrogenase, ptA: phosphate acetyltransferase, LdhA: lactate dehydrogenase, Pox: pyruvate oxidase, Gnd: 6-phosphogluconate dehydrogenase, TdcE: 2-ketobutyrate formate-lyase/pyruvate formate lyase, FHL-1: formate hydrogen lyase system, PFL: pyruvate formate lyase, GlpK: glycerol kinase, GldA: glycerol dehydrogenase, GlpABC: glycerol 3P-dehydrogenase (anaerobic), and GAPDH: glyceraldehyde 3-phosphate dehydrogenase. The metabolite intermediates are: DHA: dihydroxyacetone, DHAP: dihydroxyacetone phosphate, G3P: glycerol 3-phosphate, GA3P: glyceraldehyde 3-phosphate, Fruc 1,6-BP: fructose 1,6 bisphosphate, Fruc-6P: fructose 6-phosphate, Gluc-6P: glucose 6-phosphate, 6-PGA: 6-phosphogluconolactone, and 6PG: 6-phosphogluconate.

Fossil fuels have become an environmental problem that has prompted the search for more sustainable energy. Hydrogen (H_2_) has great potential as a clean energy source because it is a renewable, efficient, abundant, and portable form of energy. All this makes biohydrogen—hydrogen produced by dark or photofermentation—an attractive alternative [8], especially if the carbon source used to feed the microorganisms does not compete with food such as fruit and vegetable wastes, agricultural residues, or industrial effluents [9]. Among the most important microorganisms for H_2_ production by dark fermentation are facultative anaerobes such as *Escherichia coli*. This species of bacteria can consume glycerol—a subproduct of the biodiesel industry—to produce ethanol and hydrogen, having the added advantage of being one of the most commonly used species for metabolic engineering and industrial applications [10,11]. *E. coli* assimilates glycerol by conversion to dihydroxyacetone phosphate (DHAP), a glycolysis metabolite that can be transformed into pyruvate. Then, pyruvate is transformed into formate and acetyl-CoA by pyruvate formate lyase (PFL) under anaerobic and microaerobic conditions [12]. H_2_ synthesis from formate is an anaerobic process that involves the protein complex formate hydrogen lyase (FHL-1) (composed of Hyd-3 and Fdh-H), although there are two other complexes that can carry out H_2_ synthesis and oxidation (hydrogenases Hyd-1 and Hyd-2). The operation mode of these complexes (H_2_ synthesis or oxidation) strongly depends on pH and the carbon source available [13,14]. On the other hand, acetyl-CoA is transformed into acetaldehyde and ethanol by alcohol dehydrogenase (AdhE) enzymes [15] (Figure 1). 

The natural capability of *E. coli* for producing H_2_ and ethanol has been improved by implementing different strategies [11]. In our previous study, we screened 150 *E. coli* single-knockout mutants obtained from the Keio Collection [16] to identify possible beneficial phenotypes for enhanced H_2_ and/or ethanol production, using glycerol as the main carbon source [17]. We identified several novel mutants (chiefly, *gnd* and *tdcE* deletions) that were combined with other mutations previously described, such as Δ*ldhA* (deletion of the lactate dehydrogenase gene) and Δ*frdBC* (deletion of the fumarate reductase subunits B and C genes), in order to enhance H_2_ and/or ethanol yields. More specifically, the *gnd* mutant (defective 6-phosphogluconate dehydrogenase gene involved in the oxidative Pentose Phosphate Pathway) was combined with the Δ*ldhA* and Δ*frdBC* to avoid the synthesis of lactate and succinate, respectively (Δ*ldh*Δ*gnd*Δ*frdBC*::kan, designated as M4 mutant), and the deletion of the *tdcE* (2-ketobutyrate formate-lyase/pyruvate formate lyase gene, involved in threonine degradation and the synthesis of formate and acetyl-CoA) was included in the quintuple mutant (Δ*ldh*Δ*gnd*Δ*frdBC*Δ*tdcE*::kan, designated as M5). The H_2_ molar yield obtained from these engineered strains indicated that the metabolism of the carbon source was shunt towards formate in the *gnd* and *tdcE* single mutants, the M4, and, especially, the M5 strain, in which formate was increased by 2-fold compared to the wild-type strain and by 1.5-fold with respect to the *gnd* mutant. In the M5 mutant, although the specific production of H_2_ and ethanol was lower than that in the M4 mutant and wild-type strains, the H_2_ molar yield in M5 was significantly higher (by up to 1.33-fold at 94 h), as well as the ethanol molar yield (by 1.41-fold at 22 h), with respect to the wild-type and M4 strains. The lack of the *gnd* gene can increase the pool of 6-phosphogluconate (6-PG), which would lead to an increase in glyceraldehyde 3-phosphate (GA3P) and phosphoenol pyruvate (PEP) by the Entner-Doudoroff pathway, which feeds the bottom half of glycolysis [17].

This could mean that the glycerol consumption was lower but the metabolization of this carbon source was more efficient in the M5 mutant than in the wild-type and M4 strains. In this sense, the M5 mutant could be an interesting genetic background for further studies and optimization in which the excess of formate may be converted into H_2_ [17].

Other metabolic engineering strategies have also been employed to divert the carbon flux towards acetyl-CoA and formate production. One of these strategies is the redirection of the C-flux in the TCA reductive branch by evading succinate production. For instance, the deletion of the putative C4-transporter *dcuD* increased H_2_ and ethanol yields [18]. In another approach, the overexpression of the heterologous hPEPCK-M, aimed at redirecting oxalacetate (OAA) towards phosphoenolpyruvate (PEP), increased the H_2_ and ethanol specific productions and glycerol consumption [19]. In another strategy, the C-flux was efficiently redirected from malate to H_2_ by the co-overexpression of the malate dehydrogenase (Mdh) and the malic enzyme (MaeA) in the *dcuD* and *frdCdcuD* mutants [20]. 

Following our previously reported findings, in this study, we have employed metabolomics approaches, using Fourier Transformed Infrared (FT-IR) spectroscopy and Gas Chromatography–Mass Spectrometry (GC-MS) analysis, to investigate further and compare the M4, M5, and wild-type *E. coli* strains in order to identify potential metabolic bottlenecks that may affect H_2_ and ethanol synthesis, using glycerol as the carbon and energy source.

## 2. Results

Our previously engineered mutant strains, Δ*ldhA*Δ*gnd*Δ*frdBC*::FRT (M4) and Δ*ldhA*Δ*gnd*Δ*frdBC*Δ*tdcE*::FRT (M5) [17] (Table 1), displayed improved H_2_ and ethanol molar yields using a glycerol-based culture medium. However, these strains did not improve the titer and productivity with respect to the wild-type strain. However, metabolomics has been proven as an effective approach to elucidating metabolic bottlenecks that could help to design novel metabolic engineering strategies by additional gene deletions and/or overexpressions in the M5 strain. To this end, metabolomics analyses were carried out to investigate the M4, M5, and wild-type strains in order to compare their metabolic profiles and direct potential future engineering strategies. 

### 2.1. Metabolic Profile by Fourier Transformed Infrared (FT-IR) and GC-MS in the M4, M5, and Wild-Type Strains

**Figure 2:**
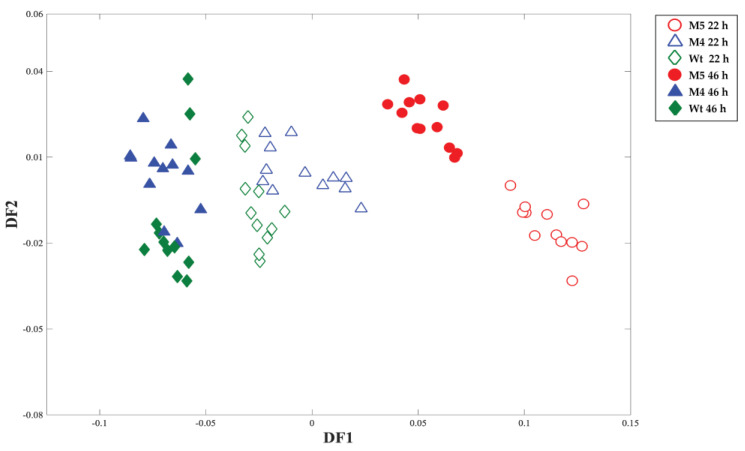
PC−DFA scores plot of the FT−IR spectral data obtained using M4, M5, and wild−type strains collected at 22 and 46 h. To extract the significant vibrations contributing to the detected clusters, DF1 and DF2 loadings are also shown in Supplementary Materials Figure S1.

FT-IR spectroscopy is a metabolic fingerprinting technique that can provide a general overview of the biochemical changes and phenotypic differences between the strains of interest. The Principal Component–Discriminant Function Analysis (PC-DFA) scores plot of the FT-IR spectral data displayed a clear separation between M5 and the other strains (M4 and wild-type) at 22 and 46 h, according to the DF1 axis (Figure 2). The DF1 loadings plot (Figure S1) illustrated the most significant vibrations that contributed to the clustering patterns observed and the separation of the M5 strain from the M4 and wild-type strains; these include 1030, 1119, 1165, 1242, 1549, 1701, 2853, and 2924 cm^−1^. The main functional groups contributing to the observed clustering patterns according to the vibrational band assignments that are related to the biological system are alcohols, amides, amines, carbo-acids, esters, and phosphorus compounds (Supplementary Materials Table S1). It is remarkable that amides and amines are found in amino acids and proteins, and carbo-acids in the C4/C6 compounds from the TCA cycle, esters in fatty acids, and phosphorus compounds can be associated with nucleotides, phospholipids, and energetic metabolism, among others. 

**Figure 3:**
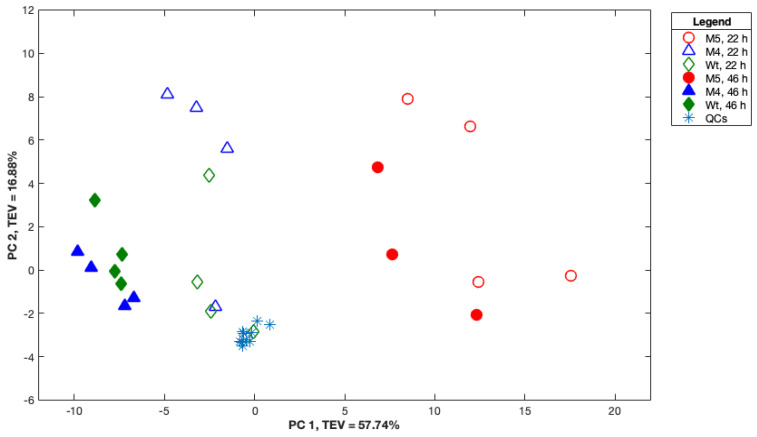
Scores plot of principal component analysis (PCA) using PC1 and PC2 of 333 peaks’ areas identified in GC−MS from M4, M5, and wild-type strains’ biomass collected at 22 and 46 h. QCs: quality control samples. TEV: total explained variance.

Although the FT-IR spectroscopy findings have clearly allowed for the discrimination of the M5 strain from the M4 and wild-type strains at two timepoints, it does not have the specificity and sensitivity required for the identification of the significant metabolites in such a complex matrix; thus, to complement these findings, GC-MS analysis was also employed in parallel to allow for a better understanding of the metabolic changes in these strains. The GC-MS raw data obtained for the M4, M5, and wild-type strains at two timepoints consisted of 333 metabolite features, which were subjected to PCA (Figure 3) to allow for the discrimination of the strains and to identify the metabolic differences in these strains. The PCA scores plot of the GC-MS data (Figure 3) displayed a clear separation of the M5 strain from the M4 and wild-type strains at both timepoints (22 and 46 h), according to the PC1 axis, with a total explained variance (TEV) of 57.74%. The GC-MS findings (Figure 3) were in complete agreement with the clustering patterns observed using the FT-IR spectral data (Figure 2). It is also worth noting that the quality control (QC) samples are clustered very tightly at the center of the PCA scores plot (Figure 3), indicating the reproducibility and validity of the sample preparation as well as the analysis methodologies. For this, in the PC1 and PC2 loadings plot, those peaks with higher PC1 loading scores (indicated with arrows in Supplementary Materials Figure S2a) were selected. In two of them, the match factor was below 50, and therefore, these compounds were not taken into account. In contrast, it was found that Methyl Dopa and 5-aminovaleric acid showed a match factor higher than 75. Then, a box plot of these two compounds was performed, although no significant differences were found in M4 and M5 with respect to the wild-type strain (Supplementary Materials Figure S2b), and they were not matched as natural *E. coli* metabolites. On the other hand, the dots plotted at the most negative scale of the PC1 axis did not show any clear separation that could allow for discriminating them as groups of compounds. It is not feasible, therefore, to determine the compounds that explain the separation of the M5 strain with respect to the M4 and wild-type strains in the PC1 axis.

### 2.2. Metabolome Overview Using the EcoCyc Omics Dashboard Tool

While PCA using the 333 peak-areas is a very useful tool for obtaining a metabolite fingerprinting, it is also interesting to have a deep insight into which metabolic pathways have been more significantly rewired in the M5 compared to the wild-type strain in order to explain the separation of M5 as observed in the PCA plot. First, those compounds with a match factor higher than 75% were selected among the 333 identified by GC-MS, resulting in a list of 160 (Supplementary Materials Table S2). This list was further refined by removing redundant compounds and those with no match in the EcoCyc Database, leading to a final selection of 78 metabolites (Supplementary Materials Table S3). The average of the metabolite areas obtained in the M4 and M5 strains was relativized with respect to the wild-type strain values and was analyzed in the Omics dashboard in the EcoCyc Database (Supplementary Materials Table S3). This tool allows for a metabolome overview that helps to explain which of the metabolic pathway fluxes could be up- or downregulated in the M5 with respect to M4 and the wild-type strain. The dashboard of this tool shows the average of the relativized data as described in the Materials and Methods section, divided into five panels that represent a system of cellular function or pathway categories: biosynthesis, degradation, energy, other pathways, and metabolites not present in the subsystem (Supplementary Materials Figure S3). In each panel, the data were ordered from left to right, showing those pathways with more differences between the groups. Each metabolic pathway was visualized by zooming in on the dashboard, which allowed for identifying several differences between the groups, especially between the M5 and the M4 with respect to the wild-type strain. For instance, it was identified that there was an enhancement of glutamate, gluconeogenesis, and fatty acid biosynthesis in M5 (Supplementary Materials Figure S4a), as well as amines, polyamines, and amino acid degradation, including increasing putrescine and allantoin at 46 h (Supplementary Materials Figure S4b). 

**Figure 4:**
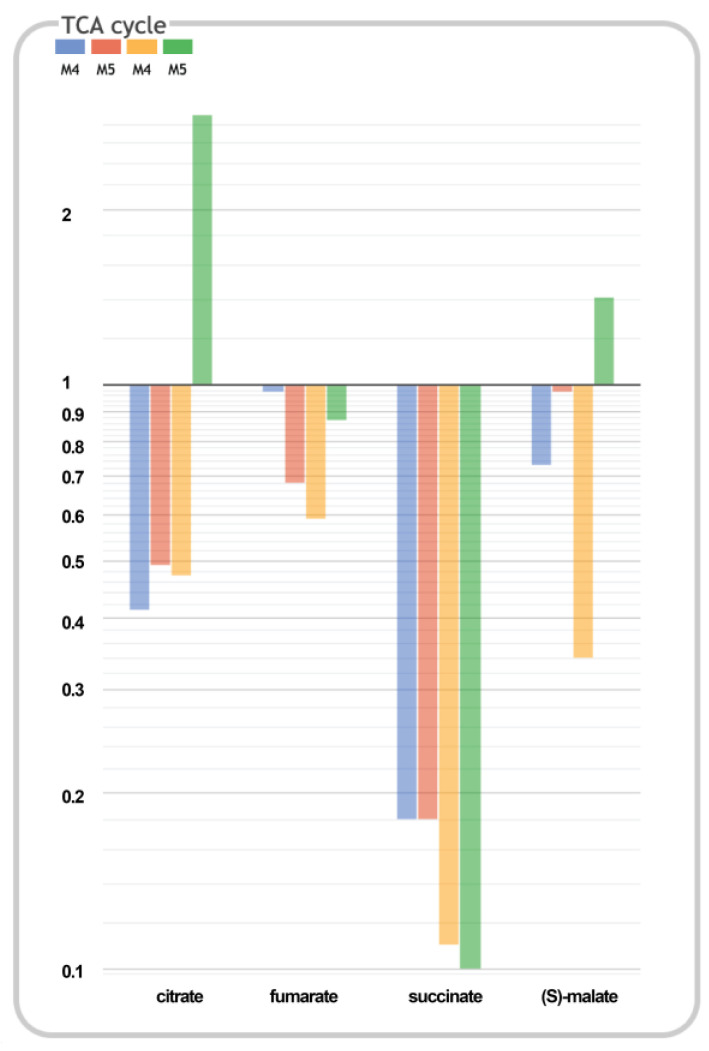
Bar graph of the relativized average of the M4 (blue and yellow) and M5 (red and green) peak areas (*n* = 4) with respect to the wild-type strain values from the biomass collected at 22 h (blue and red) and 46 h (yellow and green). Relativized values are shown in log_10_ scale.

In contrast, in M5 at 46 h, the biosynthesis of the carboxyl carrier protein, phospholipids, glycogen, and glycerol degradation as well as the synthesis of 1,2-propanediol were downregulated (Supplementary Materials Figure S4a,b). It is important to highlight that, in the TCA pathway class (energy pathway category), fumarate, succinate, malate (C4 metabolites), and citrate (C6 metabolite) were detected as equal or depleted in M4 and M5 with respect to the wild-type strain at both timepoints, with the exception of citrate and malate in M5 at 46 h, which were higher with respect to wild-type ones (Figure 4). 

**Figure 5:**
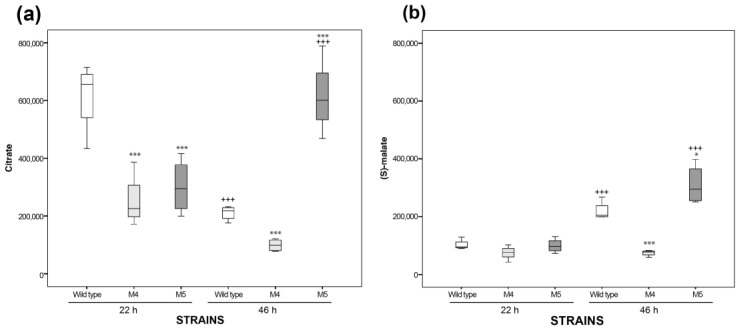
Box whiskers plots of intracellular metabolites peak areas obtained by the GC-MS of: citrate (**a**) and malate (**b**) obtained in the M4 (clear grey), M5 (dark grey), and wild-type (white) strains’ biomass collected at 22 and 46 h (*n* = 4). In asterisks, statistically significant differences are shown with respect to wild-type strain values with a *p*-value < 0.05 (*) and <0.001 (***). In crosses, the statistically significant differences of the same strain between 22 and 46 h with a *p*-value < 0.001 (^+++^) are shown.

The statistical analysis revealed significant differences for two compounds. The citrate levels in M4 and M5 at 22 h were significantly lower with respect to those in the wild-type ones; however, in M5, they were significantly higher at 46 h with respect to those of the wild-type and M5 strains at 22 h (Figure 5a). The malate concentrations were not significantly different between the strains at 22 h, but at 46 h, they were significantly increased in M5 with respect to those of the wild-type and M4 strains at 22 h (Figure 5b). In contrast, the fumarate levels were not statistically significantly different in M4 and M5 with respect to the wild-type values at both timepoints; nonetheless, the succinate levels were depleted in M4 and M5 with respect to the wild-type values, as can be expected, because the fumarate reductase B and C subunits (*frdBC*) genes had been deleted in both mutants (Supplementary Materials Figure S5). Therefore, citrate and malate were key TCA cycle metabolites that could be considered as potential sources for redirecting the C-flux in order to enhance the H_2_ and ethanol production.

### 2.3. Evaluation of H_2_ and Ethanol in M5 Mutant Strains Overexpressing Citrate Lyase (CIT) and Co-Overexpressing PEPCK or MaeA

**Figure 6:**
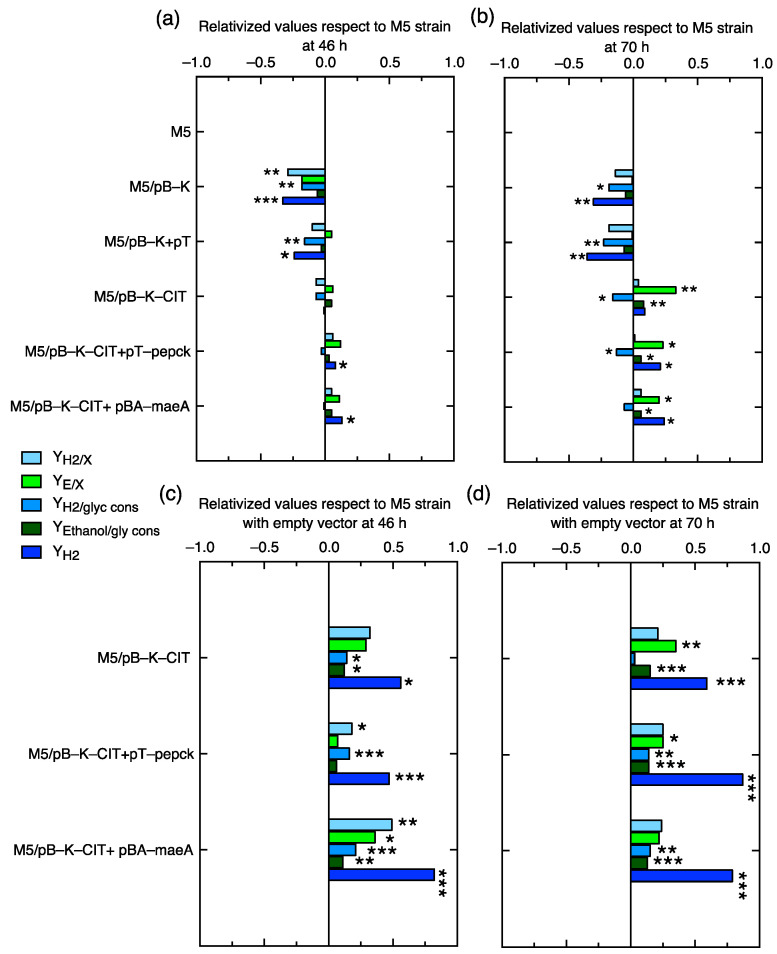
Relativized bar charts of enzyme overexpression in M5 with respect to the reference strains for the comparison of the following parameters: specific hydrogen production, Y_H2/X_ (filled in clear blue); specific ethanol production, Y_E/X_ (filled in green); hydrogen molar yield with respect to glycerol consumed, Y_H2/G_ (filled in blue); ethanol molar yield with respect to glycerol consumed, Y_E/G_ (filled in dark green); and hydrogen titer, Y_H2_ (filled in dark blue), evaluated in terms of the overexpression of citrate lyase operon (CIT), human phosphoenol pyruvate carboxykinase (hPEPCK), or malic enzyme (MaeA) in the M5 mutant relativized with M5 (**a**,**b**) or relativized with respect to M5 harboring the pBAD-18-Kan (pBK) and/or pTrc99a (pT) empty vectors (**c**,**d**) at the timepoints of 46 h (**a**,**c**) and 70 h (**b**,**d**). The inducer concentrations added were: 0.0002% (p/v) (0.013 mM) L-arabinose and/or 0.01 mM IPTG. In asterisks, the bar charts whose values are statistically significantly different with respect to the reference strain values with a *p*-value < 0.05 (*), <0.01 (**), and <0.001 (***) are shown.

Based on the metabolomics results, a promising metabolic engineering strategy could be the metabolic redirection of C-flux in the reductive and oxidative branches of the M5 mutant by the overexpression of the citrate lyase enzyme (CIT) for converting citrate into oxalacetate (OAA) and acetate and, also, the redirection of malate towards pyruvate and, subsequently, to acetyl-CoA + formate by the overexpression of anaplerotic enzymes such as hPEPCK or the malic enzyme MaeA (Figure 1). Ethanol- and hydrogen-specific productions (Y_E/X_, Y_H2/X_) and molar yields (Y_E/G_, Y_H2/G_) were evaluated in these novel strains and relativized to the M5 mutant values at 70 h in order to evaluate the metabolic rewiring after 46 h. The analysis of these parameters in the M5 strains harboring the empty vectors used to overexpress CIT, hPEPCK, and MaeA revealed a negative impact of both (pTrc99a and/or pBAD-18-Kan) vectors on most of the parameters at both timepoints (Figure 6a,b and Supplementary Materials Table S4a,b). Nonetheless, the (co)overexpression in the three M5 strains showed that Y_E/X_, Y_E/G,_ and the H_2_ titer were significantly higher with respect to M5 at 70 h. In contrast, the specific H_2_ production was not significantly different, and Y_H2/G_ was even lower in terms of the overexpression of CIT and the co-overexpression with PEPCK at 70 h (Figure 6b). As can be observed, the empty vector has a negative metabolic effect in this strain, so in order to find out the real effect in C-redirection, the values of the overexpressing strains were relativized with respect to those obtained in the M5 mutant harboring the corresponding empty vector(s) (Figure 6c,d). The values of all of the parameters evaluated were statistically significantly higher with respect to the respective reference strain in most of the cases. It can be clearly observed how the overexpression of CIT increases some of these parameter values. Furthermore, it has a synergistic effect when it is overexpressed with hPEPCK or the malic enzyme MaeA. Y_H2/X_ was statistically significantly higher at 46 h in M5 when CIT and MaeA were co-overexpressed, by 1.49-fold, while the H_2_ molar yields were significantly higher in both co-overexpressions at both timepoints. Specific ethanol productions were significantly higher in the overexpression of solely CIT at 70 h and in the co-overexpression with PEPCK by 1.25-fold at 70 h and with MaeA by 1.36-fold at 46 h. Ethanol molar yields increased significantly—between 1.13 and 1.15—in the three strains at 70 h. It is worth noting that despite the negative effect of plasmid vectors, ethanol production and ethanol yields were increased in M5/pBK-CIT+pT-pepck (97.02 mmol/g CDW and 1.24 mmol/mmol glycerol consumed) with respect to the wild-type strain values (93.34 mmol/g CDW and 0.92 mmol/mmol glycerol consumed), as referenced in Valle et al. [17]. The H_2_ titer was statistically significantly higher in the three strains at two timepoints and in the case of double CIT and PEPCK overexpression by up to 1.87-fold at 70 h and co-overexpression with MaeA by up to 1.82-fold at 46 h. 

These analyses have been complemented with the scatter plots of these parameters in the mutant strains with and without overexpressions of CIT together with PEPCK or MaeA in order to compare the profiles along the time (data included in Supplementary Materials Table S4. As can be observed, the H_2_ and ethanol specific productions in the three mutants with enzymes’ overexpressions were significantly higher with respect to the reference strains at 46 h, although these differences are more significant at 70 h, reaching ~45 mmol H_2_/gCDW and around 95–105 mmol ethanol/gCDW (Supplementary Materials Figure S6a,b). The titers of the remaining glycerol in the culture media were lower in the strains with overexpressions, which means that there is a significant increase in glycerol consumption, especially at 70 h, despite the fact that the biomass growth diminished at 70 h (Supplementary Materials Figure S6c,d, respectively). However, some glycerol still remained in the culture media, as previously observed by Valle et al. [17]. The conversion of glycerol into ethanol and H_2_ (Supplementary Materials Figure S6e,f), referred to as molar yields, is more efficient in the strains with enzymes’ overexpressions, in which the values are significantly higher with respect to those obtained in the reference strains at 70 h. 

### 2.4. Succinate, Acetate, and Glycerol Assessment in the M5 Mutant without or with the Overexpression of Citrate Lyase (CIT) and/or the Co-Overexpression of PEPCK or MaeA

**Figure 7:**
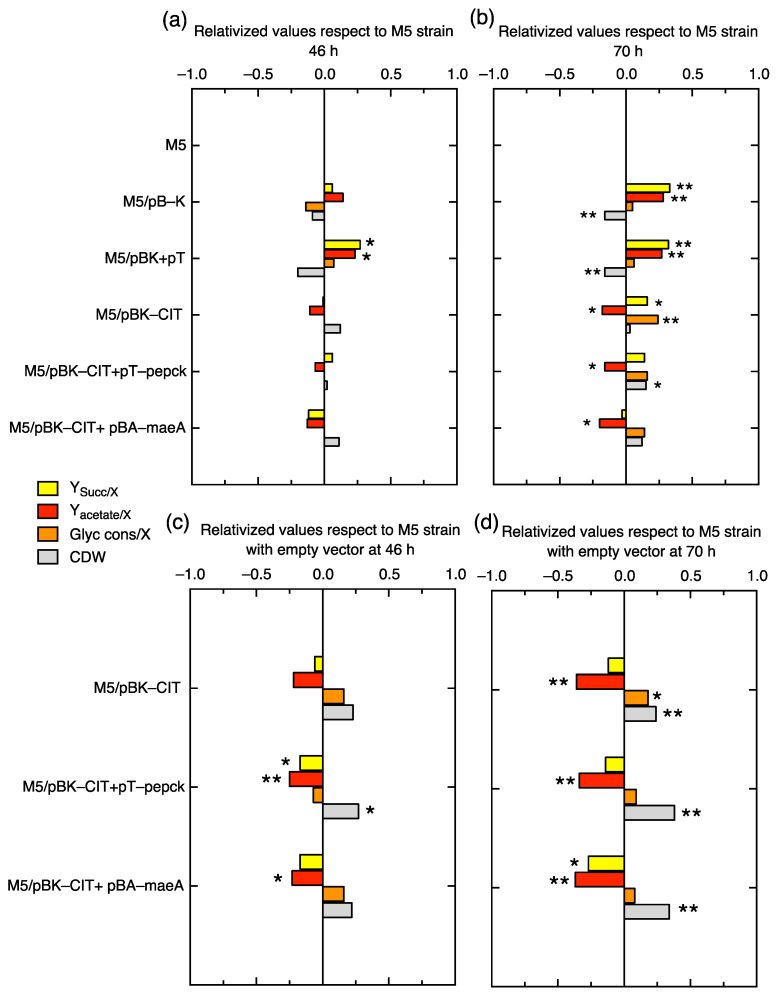
Relativized bar charts of enzyme overexpression in M5 with respect to the reference strains for the comparison of the following parameters: specific succinate efflux, Y_Succ/X_ (filled in yellow); specific acetate efflux, Y_acetate/X_ (filled in red); specific glycerol consumed, Y_G/X_ (filled in orange); and cell dried weight per liter (CDW/L) (filled in grey), evaluated in terms of the overexpression of citrate lyase operon (CIT), human phosphoenol pyruvate carboxykinase (hPEPCK), or malic enzyme (MaeA) in the M5 mutant relativized with M5 (**a**,**b**) or relativized with respect to M5 harboring the pBAD-18-Kan (pBK) and/or pTrc99a (pT) empty vectors (**c**,**d**) at the time points of 46 h (**a**,**c**) and 70 h (**b**,**d**). The inducer concentrations added were: 0.0002% (p/v) (0.013 mM) L-arabinose and/or 0.01 mM IPTG. In asterisks, the bar charts whose values are statistically significantly different with respect to the reference strain values with a *p*-value < 0.05 (*) and <0.01 (**) are shown.

Extracellular succinate and acetate were also evaluated in order to further complement the carbon output of extracellular metabolites and the carbon input with glycerol consumption in order to infer the C-flux redirection towards H_2_ and ethanol production. The succinate and acetate specific effluxes in M5 with empty vectors increased significantly at 46 h in M5/pBK+pT and also M5/pBK at 70 h (Figure 7a,b; Supplementary Materials Table S4a,b). These results are the opposite of the H_2_ and ethanol parameter’s values (Figure 6a,b). In contrast, the (co-)overexpression of CIT decreased acetate production, and glycerol consumption increased at 70 h; this effect is not observed when it is co-overexpressed with PEPCK or MaeA. Additionally, the empty vectors have an effect in bacterial growth that was significantly lower in both M5 strains with empty vectors at 70 h (Figure 7b). 

When these overexpression values were relativized with respect to the values in the M5 strain harboring the empty vectors, it was found that acetate decreased with statistically significant differences, especially in M5, with double overexpression at 46 h, and in the three M5 strains (around 1.34–1.37-fold) at 70 h (Figure 7c,d). This fact could explain how, at this timepoint, the H_2_ and ethanol yields as well as the productivities increased while succinate and acetate diminished. Glycerol consumption is not significantly different with respect to the reference strains, with the exception of CIT overexpression at 70 h, which increased by only 1.18 times (Figure 7d). The biomass growth of the co-overexpression of CIT and PEPCK significantly increased by 1.27-fold at 46 h and also in the three mutants by between 1.24- and 1.38-fold with respect to M5 with empty vectors at 70 h. Therefore, these overexpressions have improved the bacterial growth, indicating that they have an opposite effect when M5 contains empty vectors.

## 3. Discussion

The present work is based on a previous study published by Valle et al. [17] in which a high-throughput screening of single knockout strains from the *E. coli* KEIO collection was carried out to investigate the metabolic rewiring towards H_2_ and ethanol production. It was reported that the Δ*ldhA*Δ*gnd*Δ*frdBC*::FRT (M4) and Δ*ldhA*Δ*gnd*Δ*frdBCtdcE*::FRT (M5) multiple *E. coli* mutant strains, which combined some of the most favorable deletions, increased the H_2_ and ethanol molar yields. Some of these mutations were already described in the literature, such as lactate dehydrogenase (*ldhA*) or fumarate reductase (*frdBC*) deletions, which are involved in the synthesis of the competitive metabolites lactate and succinate, respectively, but other deletions like the 6-phosphogluconate dehydrogenase (*gnd*) or 2-ketobutyrate formate-lyase/pyruvate formate lyase (*tdcE*) genes were described for the first time as having a significant impact on H_2_ and ethanol production. However, although the M5 strain showed higher H_2_ and ethanol molar yields with respect to consumed glycerol, the specific H_2_ and ethanol productions were significantly lower than those in the wild-type strain. These results indicate that the M5 strain converts glycerol more efficiently towards H_2_ and ethanol but that the glycerol assimilation is the worst; moreover, it is noteworthy that the glycerol consumption and growth were significantly depleted because the gene deletions affect biological processes such as fermentation and energy derivation by the oxidation of organic compounds. These findings revealed that the metabolism is rewired to maintain the energy balance that affects the growth and glycerol consumption [17]. 

The interest in further investigating the M5 strain is that, although it shows a low growth rate, glycerol is still consumed during the stationary phase, which makes its cultivation feasible in a fed-batch bioreactor since a low biomass and a long stationary phase are advantageous in the bioreactors operation mode. However, the multiple mutations in M5 have modified its metabolic network, resulting in the undesirable features described above. In this sense, metabolomics is a feasible platform for identifying metabolic bottlenecks and thereby allowing for the estimation of the upregulation and downregulation of metabolic pathways by measuring relative levels of metabolites. This information could be very useful in designing novel metabolic engineering strategies by additional gene deletions or overexpression in the M5 strain. 

The comparison of the M5 mutant with respect to the M4 and wild-type strains using complementary techniques such as FT-IR spectroscopy and GC-MS revealed significant differences in the metabolic profiles that could be readily observed in both PCA scores plots (Figure 2 and Figure 3). The FT-IR analysis indicated the main functional groups that have been significantly changed in M5, but unfortunately, the PCA from GC-MS did not reveal the main compounds that could explain the separation of M5 metabolic fingerprinting (Supplementary Materials Figure S2). Following this, and after refining the list of compounds matched as metabolites in the EcoCyc database, the metabolic pathways that were significantly different between the groups (M4 and M5 with respect to the wild-type) were revealed. As a result, it was found that, in the M5 strain, the biosynthesis of some amino acids (histidine, cysteine, and glutamate) was upregulated at 46 h, which is in accordance with the changes observed in the amides and amines functional groups disclosed by FT-IR. Fatty acid biosynthesis was also augmented, as indicated by the increment of palmitoleate, although the phospholipids biosynthesis can be decreased considering a diminution of diphosphate (or pyrophosphate) in M5 at 46 h (Supplementary Materials Table S3 and Figure S4a); these compounds are also in agreement with the phosphorus compounds and esters classification found by FT-IR spectroscopy. Regarding carbohydrate metabolism (identified as the carbo-acids group by FT-IR), it was found that gluconeogenesis was upregulated (indicated by the increment of malate); the TCA cycle also increased in activity (increment of citrate and malate), but, in contrast, glycerol degradation was downregulated because the intracellular glycerol concentration was diminished by the lower consumption of glycerol from the extracellular medium, as previously reported [17]. The catabolism was also affected, with increased amine and polyamine degradations (indicated by the increment of putrescine, aminobutanoate, and allantoin), which are connected to amino acids metabolism and are also in accord with the amides and amines functional groups found by FT-IR (Supplementary Materials Figure S4 and Supplementary Materials Table S1).

The most remarkable differences between the M5 and the wild-type strain revealed by the metabolome overview are the significant increments in citrate and malate (Figure 5). Since both of these are key metabolites of the TCA cycle and significantly increased, this indicates a likely carbon leakage towards other metabolic pathways that would reveal an upregulation of the TCA. This is in agreement with the increment of glutamate detected, which is synthesized from α-ketoglutarate in the oxidative branch and correlates with the higher citrate intracellular levels of the M5 mutant. The diminution of intracellular glycerol in this mutant (Supplementary Materials Figure S4b) is correlated with the diminution of glycerol consumption (Supplementary Materials Table S4). Because of this, the increment of malate can be redirected by anaplerotic enzymes to gluconeogenesis, which could be related to the increment, for instance, of glutamate (Supplementary Materials Figure S4a). These differences in the metabolic pathways of M5 with respect to M4 and the wild-type strain are due to the deletion of the *tdcE* gene, which encodes a protein with both 2-ketobutyrate formate-lyase (KFL) and pyruvate formate-lyase (PFL) activities. KFL is part of an anaerobic pathway for the degradation of L-threonine in the reaction: 2-ketobutyrate + CoA → propanoyl-CoA + formate [21,22]. On the other hand, the PFL activity converts pyruvate + CoA → formate + Acetyl-CoA (Figure 1), an essential enzyme for redirecting the C flux towards H_2_ and ethanol. Surprisingly, the *tdcE* single mutant significantly enhances H_2_ and ethanol production, but the addition of this mutation in the M5 mutant only enhances the H_2_ and ethanol molar yields [17]. This deletion therefore may have a synergistic effect with other mutations. 

It is clear that C-flux in M5 is diverted towards citrate in the oxidative branch and towards malate in the reductive branch, so an additional strategy was carried out in this work to redirect the C-flux from both compounds towards H_2_ production. For this, the citrate lyase (CIT) operon and the anaplerotic enzymes (hPEPCK or MaeA) were overexpressed to shunt the conversion of citrate to OAA or malate, respectively. First of all, the use of empty vector(s) with inducible promoters (pBAD/His A and/or pTrc99a) has a metabolic burden per se, because these negatively affect H_2_ and ethanol production and positively affect succinate and acetate effluxes, concomitantly with a decrement in growth. This indicates that the empty vectors in the M5 strain under anaerobic conditions represent a metabolic burden in terms of plasmid replication (medium copy number), the expression of the antibiotic resistance gene, or maybe even the expression of a small peptide codified in a polycloning site (PCS). Despite this, the overexpression of CIT and PEPCK or MaeA enhanced ethanol and H_2_ titers, and the biomass growth was also increased (Figure 6 and Figure 7). In order to better understand the real effect of the overexpression of these enzymes, the backgrounds of empty vector(s) were subtracted in our analysis. After this normalization, the data indicate that the double overexpression with PEPCK or MaeA similarly enhances the parameters of Y_H2/X_ at 46 h and Y_H2_ and Y_H2/glycerol consumed_ at both times with respect to the overexpression of solely CIT, which evidences that the C-redirection from citrate → OAA → PEP or that of citrate → OAA → malate → Pyruvate are strategies with a similar impact on H_2_ and ethanol production. This fact is supported by the successful results obtained in the overexpression of PEPCK or MaeA in the also favorable *dcuD* mutant background [19,20]. Additionally, the CIT overexpression downregulates the oxidative shunt, although a minimum flux is needed in the oxidative branch. In this sense, it was previously described that deleting the enzyme genes involved in the oxidative branch [18] or deleting the citrate synthase (*gltA*) gene did not successfully enhance H_2_ and/or ethanol production [20]. Although the C redirection is evidenced when CIT is overexpressed together with enhanced glycerol consumption, a significant amount of glycerol still remained in the medium, so additional strategies for enhancing the glycerol input in an engineered strain could be implemented. 

The carbon redirection in citrate and malate is in accordance with the slight diminution of intracellular succinate (Figure 7c,d), because the *frdBC* deletion is very efficient in avoiding succinate and also acetate synthesis; however, acetate is necessary because the *ackpta* and *ackA-ptAPox* mutants (*ack*: acetate kinase; *pta*: phosphate acetyltransferase; *pox*: pyruvate oxidase genes) significantly diminished the synthesis of H_2_ and ethanol. CIT leads to OAA and acetate synthesis, and the extracellular acetate in the three overexpression strains tested was significantly lower with respect to that in the reference strains (Figure 7c,d), which indicates that acetate is probably consequently converted to Acetyl-CoA by coupling Ack and PtA [21]. Additional enzymes such as aldehyde dehydrogenase (AldB) (Figure 1) that catalyze the reaction acetate + NADPH → acetyl-CoA + NADP^+^ could also be involved in NADPH maintenance, because the reoxidation of NADPH can help to maintain glycolysis and to upregulate the pentose phosphate pathway (PPP) (oxidative branch) up to 6-phosphogluconate (6PG) and Entner-Doudoroff flux because the *gnd* gene is deleted in the M5 mutant (Figure 1). 

## 4. Materials and Methods

### 4.1. Strains and Growth Conditions

The bacterial growth and experimental procedures for metabolomics and protein overexpression in the *E. coli* BW25113 wild-type strain and the engineered Δ*ldhA*Δ*gnd*Δ*frdBC*::FRT (M4), Δ*ldhA*Δ*gnd*Δ*frdBC*Δ*tdcE*::FRT) (M5), and derivative M5 strains are listed in Table 1 and were previously described in [17]. All bacterial cells were cultured as four separate biological replicates. The assays for the H_2_ and ethanol productions were performed with a glycerol-based medium under anaerobic conditions in 12 mL crimp-top vials by inoculation with a bacterial culture previously grown in microaerobic conditions with LB-glycerol medium at pH 6.25, supplemented with kanamycin and/or ampicillin concentrations of 50 and 100 µg/mL, respectively, when appropriate [17]. For the metabolomics procedures (FT-IR and GC-MS analysis), samples of the bacterial biomass were collected at 22 and 46 h, and for protein overexpression, as described below, the samples were collected at 46 and 70 h. The timepoint at 70 h was set because more differences in the metabolic profiles between the M4 and M5 strains were found at 46 h. 

### 4.2. Cloning of the E. coli Citrate Lyase (CIT) Operon and the Overexpression of CIT, hPEPCK, and MaeA

For cloning the citrate lyase operon CitCDEFXG (5253 bp) (CIT), the codifying sequence of the whole operon, which includes the subunits C (citrate lyase synthetase), D (acyl carrier protein), E (citrate lyase β subunit), F (citrate lyase α subunit), X (apo-citrate lyase phosphoribosyl-dephospho-CoA transferase), and G (triphosphoribosyl-dephospho-CoA synthase), was amplified by PCR from the genomic DNA of the *E. coli* BW25113 wild-type strain using primers that incorporate the *Nhe*I restriction endonuclease site sequence in the 5′ primer forward and the *Sal*I restriction nuclease site sequence in the primer reverse. This PCR amplicon and the inducible plasmid pBAD-18-Kan (pBK) DNAs were digested with *Nhe*I and *Sal*I restriction enzymes and ligated with T4 DNA ligase. All DNA manipulations were performed as previously described [19]. The positive clones of the *E. coli* TOP10 strain harboring the cloning vector were checked by PCR, and the plasmids were purified and sequenced in an ABI 3730xl sequencer (Stab Vida, Lisbon, Portugal). The corroborated plasmid (pBK-CIT), together with pT-pepck (human *pepck* EST cloned in the pTrc99a vector) and pBA-maeA (malic enzyme A cloned in the pBAD/His A vector), obtained previously [19,20], were transformed in the M5 strain for co-overexpression with CIT. The pT and pBK empty vectors were also transformed in the M5 strain and were used as reference strains. All of these engineered strains were kept in 15% (*v*/*v*) glycerol and stored at −80 °C. Detailed information on the strains, primers, and plasmids used in this work is listed in Table 1. 

For the overexpression of the hPEPCK ORF, isopropyl-β-D-thiogalactopyranoside (IPTG) was used for the induction of the P_trc_ promoter. For the overexpression of MaeA ORF, and for the CIT operon, l-arabinose was used to induce the P_BAD_ promoter. The inducer concentrations of l-arabinose 0.0002% (p/v) (0.013 mM) and IPTG 0.01 mM were used as the most appropriate concentrations, as described [19,20].

### 4.3. FT-IR Fingerprint Analysis 

The bacterial biomass was collected by centrifugation at 5000× *g* for 5 min at 4 °C, followed by a washing step using sterile 0.9% NaCl solution. All washed samples were then normalized to a final OD_600nm_ of 15. Aliquots (20 µL) of the normalized bacterial slurries were spotted onto an FT-IR silicon microplate and dried in an oven (55 °C) for 30 min. 

FT-IR spectra were collected between 4000 and 600 cm^−1^ wavenumbers using a Bruker Equinox 55 infrared spectrometer (Bruker Optics Ltd., Coventry, UK), following our previously published methods [23,24]. Each sample class contained four biological replicates, and from each of the dried spots, three machine replicates were collected. All spectral data were pre-processed as described previously [24]. In brief, extended multiplicative signal correction (EMSC) [25] was used to scale the data, followed by replacing the CO_2_ vibrations (2400–2275 cm^−1^) with a linear trend. The pre-processed FT-IR spectral data were then subjected to principal components analysis (PCA) [26], followed by discriminant function analysis (DFA) [27].

### 4.4. GC-MS Metabolic Profiling 

The bacterial cells were cultured as four biological replicates. The samples (5 mL) were collected and quenched by adding 10 mL of cold (−48 °C) 60% methanol solution at 22 and 46 h timepoints. The samples were immediately centrifuged at −9 °C and 5000× *g* for 10 min to remove the supernatant. All quenched biomass samples were stored at −80 °C until further analysis. Internal metabolites were extracted using cold (−48 °C) 80% methanol solution and the freeze-thawing method, as described previously [7]. Cell extracts were transferred to new microcentrifuge tubes, and an aliquot (50 µL) from each sample was taken and combined in a new tube to be used as a quality control sample (QC). The internal standard (0.2 mg mL^−1^ succinic-*d*_4_ acid, 0.2 mg mL^−1^ glycine-*d*_5_) was added (100 µL) to all samples, followed by drying at 30 °C for 12 h using a speed vacuum concentrator (concentrator 5301; Eppendorf, Cambridge, UK). All samples were derivatized following a two-step methoxyamination, followed by trimethylsilylation, as reported in the literature [28]. GC-MS analysis was undertaken on an Agilent Technologies 7200 accurate mass Q-TOF mass spectrometer coupled with a 7890B GC and equipped as described previously [7]. The GC-MS data were first converted to mzXML [29] format, imported into an R (R Core Development Team 2015) environment, and deconvolved using the eRah package [30]. A total of 333 features were detected for statistical analysis. The peak intensities were log_10_-scaled and then subjected to PCA. For the assignation of the metabolites data in the metabolic pathways, the pathway tool omics dashboard in the EcoCyc database was used [31] for visualizing omics data (Supplementary Materials Tables S3 and S4), obtained by relativizing the average of the M4 and M5 metabolite peak areas (P) with respect to the wild-type strain values (P_mutant_/P_wild-type_) in a log_10_ scale. 

### 4.5. Other Analytical Techniques for the Analysis of Footprinting Metabolites

The quantification of the gas volume generated in the headspace was measured with the manometer Omega HHP350. The quantification of H_2_ and CO_2_ was analyzed by Gas Chromatography (GC), and ethanol, succinate, acetate, and glycerol were evaluated in the supernatant by High-Performance Liquid Chromatography (HPLC) using a LaChrom Elite^®^ VWR-Hitachi equipped with an HPX-87H organic acid column (Bio-Rad, Hercules, CA, USA). The method conditions were a 5 mM H_2_SO_4_ mobile phase at 0.6 mL min^−1^ and a column temperature of 50 °C [17]. Bacterial growth as the cell dry weight per liter (CDW/L) (0.31 g/L = 1 O.D.) was measured with a spectrophotometer according to the methods described in Valle et al. [17]. The parameters of the specific production of each compound were calculated as: mmol of target product/g CDW, which were: specific H_2_ production (Y_H2/X_), specific ethanol production (Y_E/X_), specific glycerol consumption (Y_G/X_), specific succinate efflux (Y_S/X_), specific acetate efflux (Y_A/X_), and titer of H_2_ (Y_H2_) in mmol/L. The H_2_ and ethanol molar yields (Y_H2/G_ and Y_E/G_) were calculated as the mol of the target product with respect to the mol of the glycerol consumed values. All of the engineered strain’s parameter values were relativized with the reference strains’ values that correspond to the M5 strain with and without empty vectors (Y_mutant_ − Y_reference_/Y_reference_) (the reference strain is denoted as 0). 

The distribution of continuous variables was evaluated by Shapiro–Wilk’s normality test. When the conditions for applying the parametric tests were achieved, Student’s *t* test was used to evaluate the statistical significance of the differences in those metabolite peak areas found between M4 or M5 with respect to those ones in the wild-type strain. The statistically significant differences of the parameter values in the (co-)overexpression of the CIT operon with and without the overexpression of PEPCK or MaeA in the M5 strain with respect to the M5 strain with and without empty vector(s) as reference strains were also evaluated. IBM^®^ SPSS^®^ Statistics v. 24 software was used for the statistical analysis, and the plots were generated with Pro Fit v. 7.0.19 (Quantum Soft, Uetikon am See, Switzerland) software.

## 5. Conclusions

In this work, we illustrate how metabolomics is a useful tool for disclosing metabolic bottlenecks that can lead to the implementation of additional metabolic engineering strategies. In this work, the overexpression of citrate lyase (CIT) and the co-overexpression of anaplerotic enzymes (PEPCK or MaeA) enhance the production of the compounds of interest. Regardless of the improvements achieved in this work, H_2_ production is still insufficient for managing an engineered *E. coli* strain to exploit its potential as a higher H_2_ and/or ethanol producer strain. In addition, we provide evidence that using plasmid vectors has a negative impact in the metabolic network, so an efficient C redirection needs an accurate expression at a physiological level, avoiding other negative factors such as the metabolic burden of protein expression or insufficient protein levels in anaerobic conditions [20]. The protein expression in aerobic conditions differs significantly from that in anaerobic conditions. Therefore, in order to avoid this obstacle, a future perspective for engineering more efficient H_2_ producer *E. coli* strains could consist of a controlled gene overexpression under chromosomal gene promoters instead of overexpression in extrachromosomal plasmid vectors, using a more tightly controlled expression that could be achieved by searching in a promoter library. Moreover, complementary omics data such as transcriptomic and fluxomic data could help in deeply understanding the metabolic network and proposing additional strategies for engineering strains that produce hydrogen and ethanol more efficiently. 

## Figures and Tables

**Table 1:** List of *E. coli* mutant strains. primers and plasmids used in this work.

Name	Genetic Characteristic	Reference
Strains		
**Wild type**	*E. coli K12 BW25113*	Keio Collection
**M4**	*∆ldhA∆gnd∆frdBC*::FRT	Valle et al. [17]
**M5**	*∆ldhA∆gnd∆frdBC∆tdcE*::FRT	Valle et al. [17]
**M5/pBK**	*∆ldhA∆gnd∆frdBC∆tdcE*/pBAD-A-Kan	This study
**M5/pBK+pT**	*∆ldhA∆gnd∆frdBC∆tdcE*/pBAD-Kan+pTrc99a	This study
**M5/pBK-CIT**	*∆ldhA∆gnd∆frdBC∆tdcE*/pBAD-Kan-CIT	This study
**M5/pBK-CIT+pT-pepck**	*∆ldhA∆gnd∆frdBC∆tdcE*/pBAD-Kan-CIT+pTrc99A-pepck	This study
**M5/pBK-CIT+pBA-maeA**	*∆ldhA∆gnd∆frdBC∆tdcE*/pBAD-Kan-CIT+ pBAD/His-A-maeA	This study
**Primers**		
**pBK-Cit-** ***NheI*** **-Fw**	ggg*GCTAGC*aggaggaattaaccATGTTCGGCAATGATATTTTCACCC	This study
**pBK-Cit-** ***SalI*** **-Rev**	ggg*GTCGAC*CAAGTGCTTAAATAAttaAATCTGTGC	This study
**CitD-Fw**	AGCTCGTCAAAAGACCCCCG	This study
**CitX-Rev**	CATGCGGTTGAGTAAATCGG	This study
***Kt***	CGGCCACAGTCGATGAATCC	Datsenko & Wanner [16]
**Plasmids**		
**pBK**	pBAD-18-Kan vector under control of P_BAD_ promoter induced by l-arabinose	J. Beckwith (Harvard)
**pT**	pTrc99a empty vector under control P_trc_ promoter induced by IPTG	Pharmacia
**pBK-CIT**	pBAD-18-Kan vector ligated with citrate lyase (CitCDEFXG) operon	This study
**pBA-maeA**	pBAD/His-A (ampicillin resistance) ligated with the malic enzyme (*maeA*) gene derived from *E. coli* BW25113	Valle et al. [20]
**pT-pepck**	pTrc99a (ampicillin resistance) ligated with phosphoenol pyruvate carboxykinase enzyme (Pepck) from *Homo sapiens*	Valle et al. [19]

In bold is denoted the Shine-Dalgarno (SD) or RBS (AGGAG) and additional nucleotides following. The same upstream sequence of initial codon ATG of pBAD/His A. In italic are denoted the sequence for recognition of enzyme restriction for cloning.

## Data Availability

Not applicable.

## Supplementary Materials

The following supporting information can be downloaded at: https://www.mdpi.com/article/10.3390/ijms241411619/s1.
