# Supplementary material for: Metabolomics of Escherichia coli for Disclosing Novel Metabolic Engineering Strategies for Enhancing Hydrogen and Ethanol Production
Source: Int J Mol Sci. 2023 Jul 18;24(14):11619. doi: 10.3390/ijms241411619 (PMC10380867; doi:10.3390/ijms241411619)

SUPPLEMENTARY MATERIAL

Table S1. List of the more significant wavenumber peaks (cm^{-1}) obtained in FT-IR loading plot (DF1) in the positive axis referred to in Fig S1.

Peak (cm^{-1})	Bond	Range	Intensity	Group	Classification	Mode
1030	C-O	1075-1000	strong	HO-R-OH	Alcohols	stretching
1119	C-O	1090-1125	strong	R2CH-OH	Alcohols	stretching
1119	C-O	1100-1210	strong	R3C-OH	Alcohols	stretching
1165	C-O	1100-1000	strong	HO-R-OH	Alcohols	stretching
1242	C-O	1260-1180	strong	Ph-OH	Alcohols	stretching
2853	CH	2900-2800	weak	C=CC=C-CHO	Aldehydes	stretching
1242	CC	1250-1200	medium	C-(CH3)3	Alkanes	skeletal vibration
2924	CH	3000-2900	medium	Cyclohexil	Alkanes	symmetric
2924	CH	2930-2920	medium	Ph-CH3	Alkanes	antisymmetric
1242	C-N	1200-1320	medium	R-CO-NH-CO-NHR	Amides	stretching
1242	NH	1200-1305	medium-weak	R-CO-NH-C	Amides	combination
1549	NH	1570-1515	strong	R-CO-NH-C	Amides	combination
1701	C=O	1650-1750	strong	R-CO-NH-CO-NHR	Amides	stretching
1030	C-N	1038-1022	weak	(R)3C-NH2	Amines	stretching
1119	C-N	1140-1080	weak-medium	(R)2CH-NH2	Amines	stretching
1242	C-O	1211-1320	strong	Ph-COOH	Carbo-acids	stretching
1242	C-O	1320-1211	strong	COOH	Carbo-acids	stretching
1701	C=O	1700-1725	strong	C-C-COOH	Carbo-acids	stretching
2924	O-H	3100-2900	medium-weak	C-C-COOH	Carbo-acids	stretching
1119	C-O-C	1300-1100	medium	COO	Esters	stretching
1119	C-O-C	1300-1100	strong	ClCOOC	Esters	stretching
1242	C-O-C	1100-1300	medium	COO	Esters	stretching
1242	C-O-C	1100-1300	strong	ClCOOC	Esters	stretching
1242	C-O	1160-1300	strong	C=C-COOR	Esters	stretching
1119	C-O-C	1060-1150	variable-strong	RCH2-O-CH2R	Ethers	antisymetric stretching
1242	C-O-C	1280-1230	medium	3-ring ethers	Ethers	symetric stretching
1242	C-O-C	1210-1310	variable strong	Ph-O-C	Ethers	antisymetric stretching
1030	P-O	1040-910	strong	(R)2P-OH	Phosphorus	stretching
1119	P=N	1100-1300	medium	Cyclic P=N	Phosphorus compounds	stretching
1242	P=N	1300-1100	medium-strong	Cyclic P=N	Phosphorus compounds	stretching
1242	P-O-Ph	1160-1260	strong	(R)3P=O	Phosphorus compounds	stretching

Table S2. List of 160 annotated compounds from GC-MS with match factor >75

Variable ID	Time Retention	Name.1	EcoCyc name (consensus)	Compound matched in EcoCyc	Redundant	Match Factor
186	13.4475	(-)-Epinephrine	(-)-Epinephrine	Non	Non	99.41
197	13.768	1,6-Anhydroglucose			Redundant	79.8
212	14.2804	1,6-Anhydroglucose	Lactitol		Non	77.15
39	7.1864	2-Hydroxypyridine	2-Hydroxypyridine	Non	Non	99.33
65	8.4223	3,4-Dihydroxy-L-phenylalanine (4TMS)			Redundant	86.02
164	12.4737	3,4-Dihydroxy-L-phenylalanine (4TMS)			Redundant	91.01
195	13.7153	3,4-Dihydroxy-L-phenylalanine (4TMS)			Redundant	90.04
255	16.0798	3,4-Dihydroxy-L-phenylalanine (4TMS)	L-dopa		Non	96.55
66	8.6933	4-Hydroxypyridine	4-Hydroxypyridine	Non	Non	98.06
290	17.6208	5-Aminovaleric acid	5-aminopentanoate		Non	88.46
235	15.2356	5-Hydoxytryptamine (5TMS)	Cadaverine		Redundant	81.06
321	19.801	5-Hydoxytryptamine (5TMS)			Redundant	87.39
333	21.3294	5-Methylthioadenosine	S-methyl-5'-thioadenosine		Non	94.19
179	13.0665	6-Hydroxynicotinic acid	6-Hydroxynicotinic acid	Non	Non	86.61
247	15.6664	Adenine (2TMS)	Adenine		Non	85.37
136	11.2154	Alanylalanine	Hypotaurine		Non	76.76
103	10.0902	Benzoic acid	Benzoate		Non	92.5
297	17.9881	Cadaverine (4TMS)	Cadaverine		Non	93.82
232	15.1224	Citric acid (4TMS)	Citrate		Non	85.15
44	7.4491	Creatinine	Creatinine	Non	Non	79.47
29	6.704	D-(-)-Arabinose (4TMS;1MEOX)	D-arabinose		Non	89.56
242	15.4502	D-(+)-Fucose	D-Fucose		Non	80.26
287	17.4727	D-(+)-Fucose	D-Fucose		Redundant	79.65
183	13.3331	D-3-Phenyllactic acid	Protocatechuate		Non	91.73
278	17.119	D-Arabinose-5-phosphate	D-arabinose 5-phosphate		Non	81.06
279	17.1155	D-Arabinose-5-phosphate			Redundant	78.3
24	6.3781	D-Galactosamine (5TMS)	D-galactosamine		Non	88.45
154	12.0402	D-Homoserine	D-homoserine		Non	87.64
210	14.1603	DethioBiotin	Dethiobiotin		Non	92.4
37	7.0306	DL-2,3-Diaminopropionic acid (2TMS)	DL-2,3-Diaminopropionic acid	Non	Non	96.14
201	13.96	DL-Homocysteine (3TMS)	L-Homocysteine		Non	90.33
86	9.606	DL-Norvaline	DL-norvaline		Non	97.73
304	18.7337	DL-Norvaline			Redundant	78.26

317	19.4096	DL-Norvaline			Redundant	97
188	13.5286	DL-Pipecolic acid			Redundant	97.27
237	15.2919	DL-Pipecolic acid			Redundant	89.12
259	16.2234	DL-Pipecolic acid			Redundant	97.59
271	16.9119	DL-Pipecolic acid			Redundant	97.5
272	16.865	DL-Pipecolic acid			Redundant	97.73
280	17.1564	DL-Pipecolic acid			Redundant	88.63
283	17.3511	DL-Pipecolic acid			Redundant	97.28
289	17.6387	DL-Pipecolic acid	DL-Pipecolic acid	Non	Non	98.5
293	17.7523	DL-Pipecolic acid			Redundant	95.79
294	17.791	DL-Pipecolic acid			Redundant	96.34
299	18.2635	DL-Pipecolic acid			Redundant	90.53
312	19.1198	DL-Pipecolic acid			Redundant	97.91
318	19.5122	DL-Pipecolic acid			Redundant	93.32
320	19.7515	DL-Pipecolic acid			Redundant	89.11
172	12.8043	DL-Pyroglutamic acid	5-oxo-D-proline		Non	90.53
91	9.7993	Dopamine			Redundant	98.6
105	10.2022	Dopamine	Dopamine		Non	98.98
142	11.5638	Dopamine			Redundant	98.82
147	11.707	Dopamine			Redundant	94.48
156	12.0834	Dopamine			Redundant	98.65
191	13.5939	Dopamine			Redundant	97.66
194	13.7332	Dopamine			Redundant	96.82
218	14.5786	Dopamine			Redundant	97.52
231	15.1457	Dopamine			Redundant	94.95
238	15.3373	Dopamine			Redundant	97.04
244	15.5884	Dopamine			Redundant	97.44
264	16.4116	Dopamine			Redundant	98.06
282	17.2907	Dopamine			Redundant	96.75
286	17.4317	Dopamine			Redundant	96
307	18.9179	Dopamine			Redundant	98.22
311	19.0727	Dopamine			Redundant	95.95
323	20.0659	Dopamine			Redundant	95.2
328	20.3835	Dopamine			Redundant	88.57
330	20.9505	Dopamine			Redundant	94
132	11.1082	Fumaric acid (2TMS)	Fumarate		Non	90.02
202	13.9698	Galactitol	Galactitol		Non	78.62
71	8.9401	Gallic acid			Redundant	89.21
82	9.4217	Gallic acid	Gallate		Non	89.55
182	13.2144	Glutathione disulfide (xTMS)	glutathione disulfide		Non	92.06
253	15.924	Glutathione disulfide (xTMS)	glutathione disulfide		Redundant	81.06
80	9.4145	Glycerol (3TMS)	Glycerol		Non	77.12
216	14.3706	Glycerol-2-phosphate	Glycerol 2-phosphate		Non	81.67

50	7.7507	Glycolic acid (2TMS)	Glycolate		Non	85.33
51	7.7549	Glycolic acid (2TMS)			Redundant	84.76
42	7.3599	Harmaline (1TMS)	Harmaline		Non	93.71
173	12.8292	Harmaline (1TMS)			Redundant	90.33
292	17.6973	Harmaline (1TMS)			Redundant	88.38
64	8.3982	Hydrocinnamate	3-phenylpropanoate		Non	93.09
10	5.444	Hydroxylamine (3TMS)			Redundant	79.15
11	5.4444	Hydroxylamine (3TMS)			Redundant	79.68
20	6.0345	Hydroxylamine (3TMS)			Redundant	80.11
62	8.3091	Hydroxylamine (3TMS)	Hydroxylamine		Non	91.01
316	19.3167	Icosanoic acid	Palmitate		Redundant	78.79
208	14.0884	Kojic acid	Kojic acid		Non	91.35
222	14.7929	Kojic acid			Redundant	88.58
162	12.3891	L-(-)-Malic acid (3TMS)	(S)-malate		Non	93.16
46	7.4892	L-(+)-Lactic acid	Lactate		Non	93.96
126	10.842	L-(+)-Tartarate	L-tartrate		Non	77.44
148	11.7511	L-2-Aminobutyric acid	(S)-2-aminobutanoate		Redundant	77.54
57	8.1381	L-Alanine (3TMS)	L-alanine		Non	99.83
198	13.8477	L-Alanine (3TMS)	L-alanine		Redundant	97
192	13.6667	L-Aspartic acid (3TMS)	L-aspartic acid		Redundant	85.89
153	12.0082	L-Citrulline			Redundant	78.45
234	15.2111	L-Citrulline	L-citrulline		Non	94.34
190	13.5648	L-Glutamic acid (3TMS)	L-glutamate		Non	95.6
161	12.3272	L-Glutamine (3TMS)	L-glutamine		Non	93.65
263	16.3733	L-Glutathione (xTMS)	S-hexylglutathione		Non	97.14
56	8.0894	L-Histidine (3TMS)			Redundant	94.82
96	9.9303	L-Histidine (3TMS)			Redundant	78.52
254	15.93	L-Histidine (3TMS)	L-histidine		Non	98.27
261	16.2701	L-Histidine (3TMS)			Redundant	91.49
18	5.856	L-Homocitrulline	L-Homocitrulline	Non	Non	96.31
115	10.4989	L-Isoleucine (2TMS)	L-isoleucine		Non	96.08
165	12.5062	L-Mandelic acid			Redundant	90.71
250	15.7752	L-Mandelic acid			Redundant	94.63
322	19.8796	L-Mandelic acid			Redundant	85.76
326	20.165	L-Mandelic acid			Redundant	90.32
329	20.8721	L-Mandelic acid			Redundant	96.23
332	21.2361	L-Mandelic acid	L-Mandelic acid	Non	Non	96.65
171	12.7487	L-Methionine (2TMS)	L-methionine		Non	98.9
116	10.5035	L-Norleucine (2TMS)			Redundant	97.6
152	11.9723	L-Norleucine (2TMS)	L-norleucine		Non	98
225	14.9865	L-Norleucine (2TMS)			Redundant	94.81
258	16.1383	L-Norleucine (2TMS)			Redundant	79.71
85	9.6063	L-Norvaline (4TMS)	L-norvaline		Non	96.88
233	15.1482	L-Ornithine (2TMS)	L-ornithine		Non	97.54

196	13.7127	L-Phenylalanine (2TMS)	L-phenylalanine		Non	92.32
16	5.7631	L-Prolinamide			Redundant	93.65
117	10.5958	L-Proline (2TMS)	L-proline		Non	99.7
189	13.5316	L-Proline (2TMS)			Redundant	87.83
256	16.1265	L-Proline (2TMS)			Redundant	75.95
262	16.3476	L-Proline (2TMS)			Redundant	95.25
270	16.7688	L-Proline (2TMS)			Redundant	96.03
314	19.1777	L-Proline (2TMS)			Redundant	98.87
89	9.7377	L-Pyroglutamic acid (2TMS)	5-oxo-L-proline		Non	97.81
1	5.1446	Linolenic acid (1TMS)			Redundant	76.31
2	5.1689	Linolenic acid (1TMS)	Linolenic acid	Non	Non	81.19
5	5.2047	Linolenic acid (1TMS)			Redundant	78.84
170	12.6928	Methyl Dopa	L-Aspartic acid		Non	89.64
276	17.0606	Myo-Inositol (6TMS)	Myo-inositol		Non	88.96
69	8.9157	N-Acetyl-L-Leucine	Histamine		Non	76.13
76	9.2003	N-Acetyl-L-Leucine			Redundant	82.51
73	9.0685	N-Methyl-DL-Alanine (2TMS)	(S)-2-aminobutanoate		Non	96.82
6	5.2415	N-Methylethanolamine	L-alanine		Redundant	76.62
137	11.245	Nonanoic acid(C9)	Nonanoic acid	Non	Non	87.7
13	5.4797	O-Succinyl-L-Homoserine			Redundant	85.66
224	14.922	O-Succinyl-L-Homoserine	O-succinyl-L-homoserine		Non	95.47
243	15.5024	O-Succinyl-L-Homoserine			Redundant	87.03
3	5.1959	Oxalic acid (2TMS)	Oxalate		Non	80.95
4	5.1945	Oxalic acid (2TMS)			Redundant	75.75
274	16.8518	Palmitic acid (1TMS)	Palmitate		Non	84.63
285	17.4307	Palmitoleate	Palmitoleate		Non	84.88
40	7.3012	Pentachlorophenol (1TMS)	Pentachlorophenol		Non	75.25
26	6.5008	Propyleneglycol	Propane-1,2-diol		Non	99.31
79	9.3492	Propyleneglycol			Redundant	93.88
107	10.2732	Pyrophosphate	Diphosphate		Non	90.29
109	10.2224	Pyrophosphate			Redundant	88.88
113	10.5144	Pyrophosphate			Redundant	85.16
220	14.6624	Ribulose-1,5-Bisphosphate	a [ribulose-1,5-bisphosphate-carboxylase]-lysine		Non	78.97
58	8.1406	Shikimic acid (4TMS)	Shikimate		Non	88.64
298	18.1401	Stearic acid (1TMS)	Stearate		Non	83.79
121	10.7374	Succinic acid (2TMS)	Succinate		Non	83.04
144	11.6187	Thymine (2TMS)	Thymine		Non	84.23
130	10.9949	Uracil (2TMS)	Uracil		Non	77.58
104	10.1815	Urea	Urea		Non	93.85
277	17.0604	Xylitol	Xylitol		Non	79.64

Table S3. Normalized metabolite peaks' areas of M4 and M5 mutants respect to wild type values at 22 h and 46 h. In red are highlighted the values higher than 1 and in blue the values lower than 1. The colour intensities are directly proportional to the numerical level.

Variable ID	EcoCyc name (consensus)	M4 22h	M5 22h	M4 46h	M5 46h
254	L-histidine	1.44	1.57	1.39	6.30
297	Cadaverine	1.16	2.47	1.58	3.66
216	Glycerol 2-phosphate	0.87	1.73	0.77	3.09
232	Citrate	0.41	0.49	0.47	2.91
152	L-norleucine	1.83	3.44	1.14	2.72
255	L-dopa	1.40	1.66	1.12	2.72
263	S-hexylglutathione	1.37	2.27	0.93	2.67
208	Kojic acid	2.72	2.04	2.50	2.67
182	glutathione disulfide	1.60	2.21	1.07	2.48
136	Hypotaurine	1.05	1.07	1.68	2.44
220	a [ribulose-1,5-bisphosphate-carboxylase]-lysine	0.89	1.00	0.50	2.31
233	L-ornithine	1.90	2.07	1.25	2.15
234	L-citrulline	1.57	1.82	1.10	1.97
289	DL-Pipecolic acid	1.25	1.92	0.99	1.87
290	5-aminopentanoate	1.27	1.60	1.23	1.83
190	L-glutamate	1.12	1.15	0.78	1.83
171	L-methionine	1.32	1.56	1.13	1.74
104	Urea	1.23	0.87	1.19	1.73
276	Myo-inositol	1.53	1.66	1.28	1.71
18	L-Homocitrulline	1.27	1.49	1.29	1.70
42	Harmaline	0.89	1.71	0.93	1.66
86	DL-norvaline	1.18	1.21	1.34	1.57
224	O-succinyl-L-homoserine	1.26	1.00	0.95	1.55
179	6-Hydroxynicotinic acid	0.86	1.40	1.16	1.50
57	L-alanine	1.58	1.61	1.33	1.48
162	(S)-malate	0.73	0.97	0.34	1.42
50	Glycolate	1.19	1.46	0.88	1.37
332	L-Mandelic acid	1.25	1.85	0.84	1.37
144	Thymine	0.72	1.25	0.69	1.35
210	Dethiobiotin	1.09	1.29	1.32	1.34
66	4-Hydroxypyridine	1.06	1.50	0.93	1.28
126	L-tartrate	1.32	1.28	1.07	1.24
285	Palmitoleate	0.90	1.31	0.77	1.23
115	L-isoleucine	1.18	1.15	1.12	1.23
277	Xylitol	1.49	1.86	1.33	1.21
73	(S)-2-aminobutanoate	1.23	1.27	1.00	1.20
85	L-norvaline	1.10	1.11	1.04	1.19
39	2-Hydroxypyridine	1.13	1.59	1.08	1.17
202	Galactitol	0.85	1.50	0.60	1.16
196	L-phenylalanine	1.01	1.03	1.01	1.15

89	5-oxo-L-proline	1.24	1.21	1.14	1.07
40	Pentachlorophenol	1.12	1.21	1.02	1.04
172	5-oxo-D-proline	1.03	1.01	1.00	1.03
29	D-arabinose	0.90	1.13	1.25	0.96
117	L-proline	1.45	0.83	1.18	0.94
69	Histamine	1.15	1.42	0.93	0.92
24	D-galactosamine	1.04	1.08	1.14	0.91
242	D-Fucose	1.04	1.23	0.81	0.91
132	Fumarate	0.97	0.68	0.59	0.87
298	Stearate	1.11	1.35	0.85	0.85
161	L-glutamine	1.04	1.08	0.86	0.85
274	Palmitate	1.05	1.16	0.85	0.84
2	Linolenic acid	1.43	1.02	0.59	0.81
82	Gallate	1.08	1.08	0.83	0.80
212	Lactitol	0.96	0.75	0.83	0.77
44	Creatinine	0.30	0.28	0.94	0.75
107	Diphosphate	1.06	1.25	1.11	0.73
278	D-arabinose 5-phosphate	0.54	0.97	0.43	0.72
62	Hydroxylamine	1.04	0.92	0.86	0.71
46	Lactate	0.22	0.35	1.00	0.70
103	Benzoate	0.75	0.75	0.76	0.66
58	Shikimate	1.04	1.01	0.70	0.66
247	Adenine	0.93	1.48	0.55	0.55
80	Glycerol	0.91	0.67	0.79	0.55
64	3-phenylpropanoate	1.09	1.04	0.82	0.53
201	L-Homocysteine	0.99	0.88	0.91	0.52
130	Uracil	0.96	0.73	0.71	0.49
170	L-Aspartic acid	1.04	0.98	0.29	0.48
37	DL-2,3-Diaminopropionic acid	1.05	0.76	1.21	0.48
105	Dopamine	1.04	0.51	1.01	0.47
137	Nonanoic acid	0.85	0.96	0.65	0.44
183	Protocatechuate	1.80	0.96	1.12	0.42
186	(-)-Epinephrine	0.70	1.39	0.75	0.38
3	Oxalate	0.94	0.81	0.45	0.36
333	S-methyl-5'-thioadenosine	0.64	1.39	0.49	0.34
154	D-homoserine	0.67	1.04	0.50	0.23
26	Propane-1,2-diol	0.71	0.76	1.41	0.10
121	Succinate	0.18	0.18	0.11	0.10

Table S4. Mean, standard deviation and relativized values respect to M5 strain and also respect to the M5 with empty vector(s) at 46 (a) and 70 h (b) of the following parameters: of the Specific H_2 production and ethanol prodution (mmol/gCDW), H_2 and ethanol molar yield (mmol/mmol glycerol consumed), specific succinate and acetate efflux (mmol/gCDW), specific glycerol consumtion (mmol glycerol consumed/gCDW), volumetric H_2 production (mmol/L) and growth biomass refered as cell dried weight per liter (CDW/L).

a)

		$Y_{H2/X}$				$Y_{E/X}$				$Y_{H2/glic\ cons}$				$Y_{E/glic\ cons}$				$Y_{Succ/X}$			
ID2	Strains	Mean	S.D.	Relativ empty vector	Relativ with Ref strain	Mean	S.D.	Relativ empty vector	Relativ with Ref strain	Mean	S.D.	Relativ empty vector	Relativ with Ref strain	Mean	S.D.	Relativ empty vector	Relativ with Ref strain	Mean	S.D.	Relativ empty vector	Relativ with Ref strain
990	M5	28.94	4.49	1	1.00	59.12	9.69	1	1.00	0.56	0.05	1	1.00	1.13	0.04	1	1.00	1.48	0.29	1	1.00
991	M5/pB-K	20.39	2.76	0.00	0.70	48.29	8.83	0.00	0.82	0.45	0.03	0.00	0.82	1.07	0.04	0.00	0.94	1.57	0.26	0.00	1.06
992	M5/pB-K+pT	26.06	1.86	0.00	0.90	61.85	6.83	0.00	1.05	0.46	0.01	0.00	0.83	1.10	0.05	0.00	0.97	1.89	0.22	0.00	1.27
995	M5/pB-K-CIT	27.00	6.57	1.32	0.93	62.44	15.90	1.29	1.06	0.52	0.02	1.14	0.93	1.19	0.08	1.12	1.05	1.47	0.36	0.94	0.99
999	M5/pB-K-CIT+pT-pepck	30.65	3.98	1.18	1.06	66.45	13.17	1.07	1.12	0.54	0.03	1.16	0.97	1.16	0.08	1.06	1.03	1.57	0.17	0.83	1.06
1001	M5/pB-K-CIT+ pBA-maeA	30.38	5.46	1.49	1.05	65.53	12.35	1.36	1.11	0.55	0.04	1.21	0.99	1.19	0.07	1.11	1.05	1.31	0.18	0.83	0.88

	$Y_{Succ/X}$				$Y_{acetate/X}$				Glyc cons/X				Y_{H2}				CDW (g/L)			
Strains	Mean	S.D.	Relativ empty vector	Relativ with Ref strain	Mean	S.D.	Relativ empty vector	Relativ with Ref strain	Mean	S.D.	Relativ empty vector	Relativ with Ref strain	Mean	S.D.	Relativ empty vector	Relativ with Ref strain	Mean	S.D.	Relativ empty vector	Relativ with Ref strain
M5	1.48	0.29	1	1.00	24.10	3.60	1	1.00	52.44	9.24	1	1.00	11.44	0.89	1	1.00	0.40	0.08	1	1.00
M5/pB-K	1.57	0.26	0.00	1.06	27.44	4.26	0.00	1.14	45.17	7.03	0.00	0.86	7.29	0.89	0.00	0.64	0.37	0.09	0.00	0.91
M5/pB-K+pT	1.89	0.22	0.00	1.27	29.66	3.33	0.00	1.23	56.18	4.01	0.00	1.07	8.49	1.48	0.00	0.74	0.32	0.05	0.00	0.80
M5/pB-K-CIT	1.47	0.36	0.94	0.99	21.44	5.73	0.78	0.89	52.44	9.24	1.16	1.00	11.35	1.21	1.56	0.99	0.45	0.16	1.23	1.12
M5/pB-K-CIT+pT-pepck	1.57	0.17	0.83	1.06	22.36	2.99	0.75	0.93	52.44	9.24	0.93	1.00	12.48	0.64	1.47	1.09	0.41	0.06	1.27	1.02
M5/pB-K-CIT+ pBA-maeA	1.31	0.18	0.83	0.88	21.08	4.10	0.77	0.87	52.44	9.24	1.16	1.00	13.24	1.32	1.82	1.16	0.45	0.09	1.22	1.11

b)

		$Y_{H2/X}$				$Y_{E/X}$				$Y_{H2/glic\ cons}$				$Y_{E/glic\ cons}$			
ID2	Strains	Mean	S.D.	Relativ empty vector	Relativ with wt	Mean	S.D.	Relativ empty vector	Relativ with wt	Mean	S.D.	Relativ empty vector	Relativ with wt	Mean	S.D.	Relativ empty vector	Relativ with wt
1003	M5	42.52	4.50	0.00	1.00	79.05	3.74	0.00	1.00	0.63	0.04	0.00	1.00	1.18	0.03	0.00	1.00
1004	M5/pB-K	36.43	4.54	0.00	0.86	78.16	7.51	0.00	0.99	0.51	0.02	0.00	0.81	1.10	0.01	0.00	0.94
1005	M5/pB-K+pT	34.56	2.98	0.00	0.81	77.87	5.65	0.00	0.99	0.48	0.01	0.00	0.77	1.09	0.02	0.00	0.93
1006	M5/pB-K-CIT	44.08	2.77	1.21	1.04	105.44	4.12	1.35	1.33	0.53	0.01	1.03	0.84	1.27	0.01	1.15	1.08
1007	M5/pB-K-CIT+pT-pepck	43.07	4.95	1.25	1.01	97.02	9.92	1.25	1.23	0.55	0.01	1.14	0.87	1.24	0.01	1.14	1.06
1008	M5/pB-K-CIT+pBA-maeA	45.15	3.74	1.24	1.06	95.05	7.95	1.22	1.20	0.59	0.01	1.15	0.93	1.24	0.01	1.13	1.06

	$Y_{Succ/X}$				$Y_{acetate/X}$				Glyc cons/X				Y_{H2}				CDW (g/L)			
Strains	Mean	S.D.	Relativ empty vector	Relativ with wt	Mean	S.D.	Relativ empty vector	Relativ with wt	Mean	S.D.	Relativ empty vector	Relativ with wt	Mean	S.D.	Relativ empty vector	Relativ with wt	Mean	S.D.	Relativ empty vector	Relativ with Ref strain
M5	1.82	0.07	0.00	1.00	30.09	1.20	0.00	1.00	67.22	2.98	0.00	1.00	12.91	1.29	0.00	1.00	0.30	0.00	1.00	1.00
M5/pB-K	2.41	0.19	0.00	1.33	38.36	1.59	0.00	1.28	70.79	5.95	0.00	1.05	8.89	0.58	0.00	0.69	0.25	0.01	0.00	0.84
M5/pB-K+pT	2.39	0.18	0.00	1.32	38.15	2.77	0.00	1.27	71.35	6.22	0.00	1.06	8.33	0.28	0.00	0.64	0.25	0.01	0.00	0.84
M5/pB-K-CIT	2.12	0.12	0.88	1.16	24.74	2.53	0.64	0.82	83.24	3.93	1.18	1.24	14.12	0.57	1.59	1.09	0.31	0.01	1.24	1.03
M5/pB-K-CIT+pT-pepck	2.07	0.19	0.86	1.14	25.18	2.70	0.66	0.84	78.06	7.48	1.09	1.16	15.59	0.45	1.87	1.21	0.35	0.03	1.38	1.15
M5/pB-K-CIT+pBA-maeA	1.76	0.20	0.73	0.97	24.11	3.18	0.63	0.80	76.48	6.58	1.08	1.14	15.95	0.40	1.79	1.24	0.34	0.03	1.34	1.12

Figure S1. Discriminant Function (DF) loadings respect to wavenumbers (cm^{-1}) extracted from multivariate analysis of FT-IR data performed in M4, M5 and wild type strains at 22 and 46h.

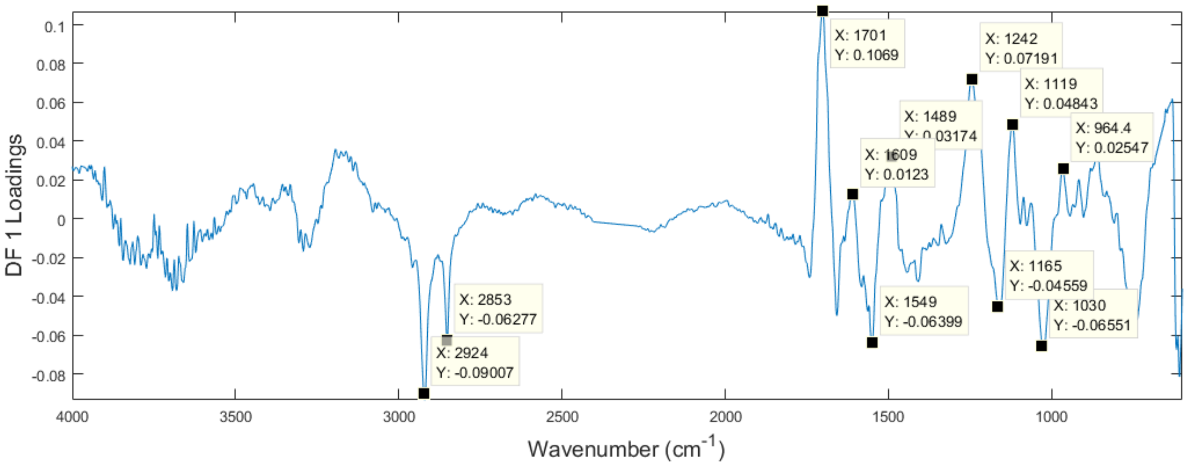

Figure S2. Plot of PC1 and PC2 loadings from GC-MS data of M4, M5 and wild type strains at 22 and 46 h (a), table of compounds with highest loading plots values remarked with arrows in the right positive of PCA and in the red the Match factor values lower than 75 and (b) box plots of compounds with match factor higher than 75, obtained from the table below the plot.

a)

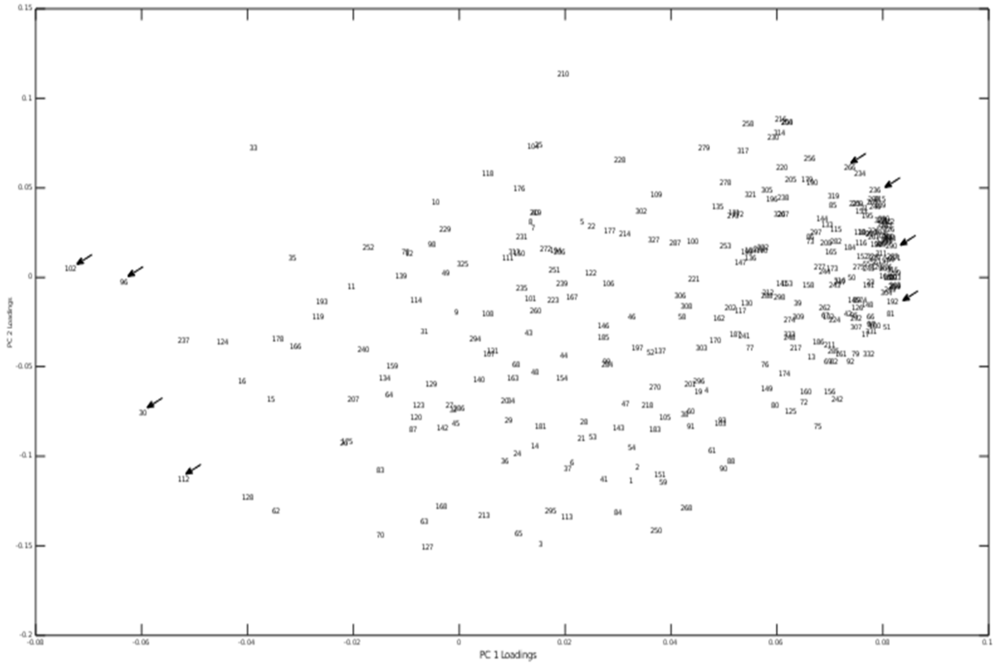

b)

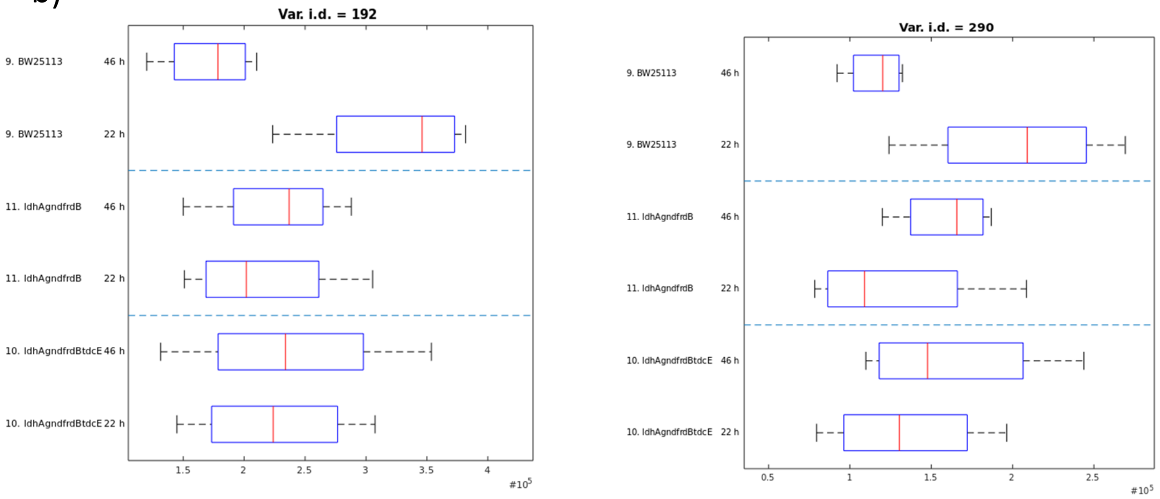

Compound ID	Retention Time	Name	Match Factor
192	13.6667	Methyl Dopa	92.24
236	15.2598	16-Hydroxyhexadecanoic acid (2TMS)	24.56
266	16.5631	L-Kynurenine	33.98
290	17.6208	5-Aminovaleric acid	88.46

Figure S3. Relativized omics data obtained from the refined list of 78 metabolites **(Supplementary material Table S3)** of M4 and M5 average values (*n*=4) respect to wild type strain ones. The set of panels, represent each one a system of cellular function, e.g. Biosynthesis. For each panel, it is shown a graph depicting omics data for each of a set of subsystems, e.g. Amino Acid Biosynthesis (AA Syn) and Carbohydrates Biosynthesis (Carbo Syn). Each panel has its own y-axis, so that omics data for the different subsystems within a panel can readily be compared with each other. Multiple timepoints or experimental conditions are plotted as separate data series within the graph. Clicking on the plot for a given subsystem brings up a detail panel, breaking that subsystem down further into its component subsystems. At the lowest level, the values along the x-axis correspond to the individual objects in the dataset in this case metabolites for metabolomics data. Each subsystem metabolic pathway is ordered in decreasing changes between series since left to right.

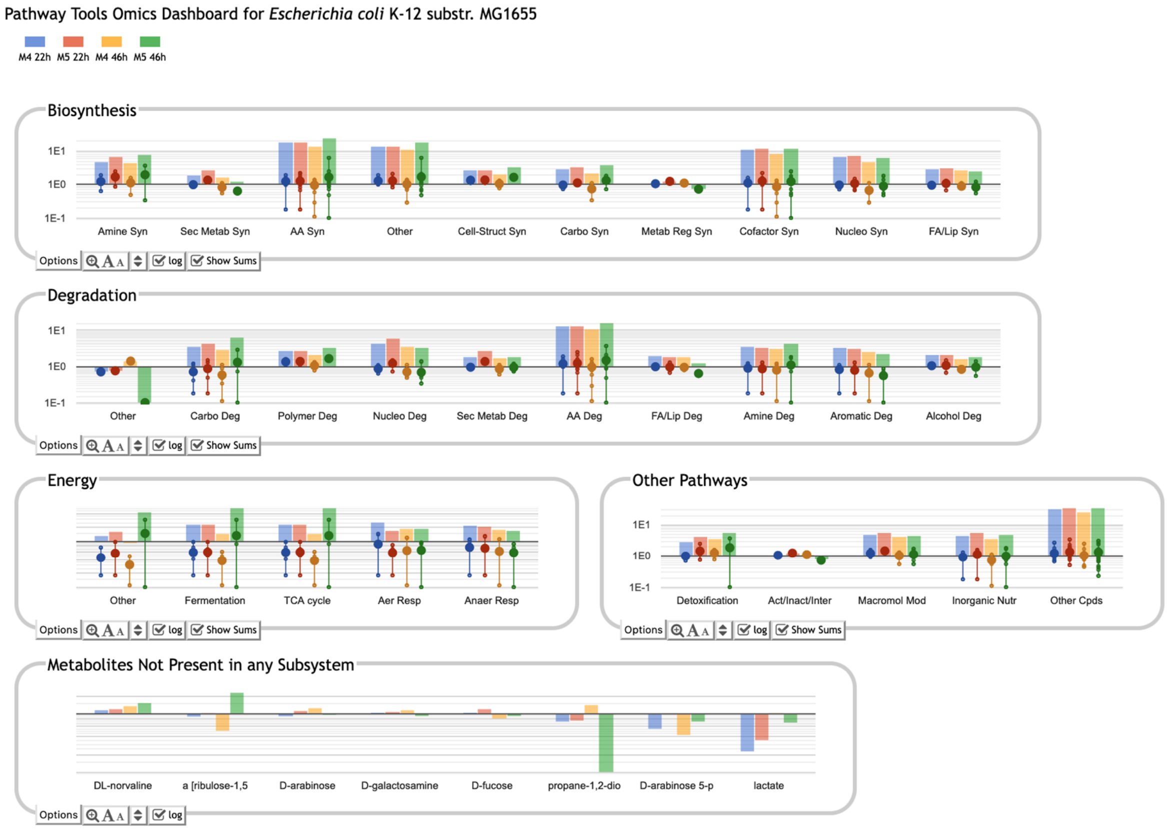

Figure S4. Subsystems obtained from each panel or metabolic pathway classes (extracted from the omics data overview in Figure S3).

a) Biosynthesis

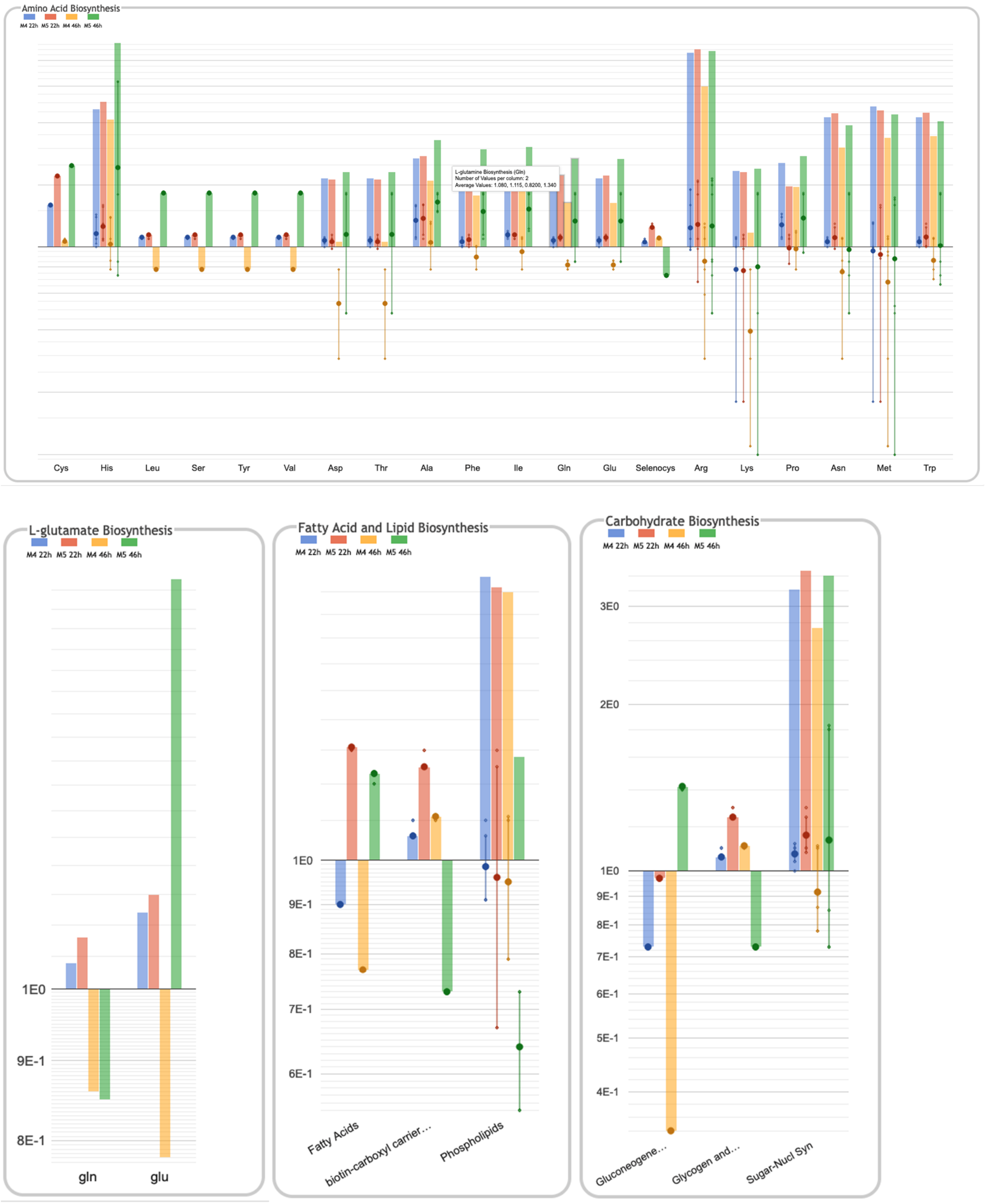

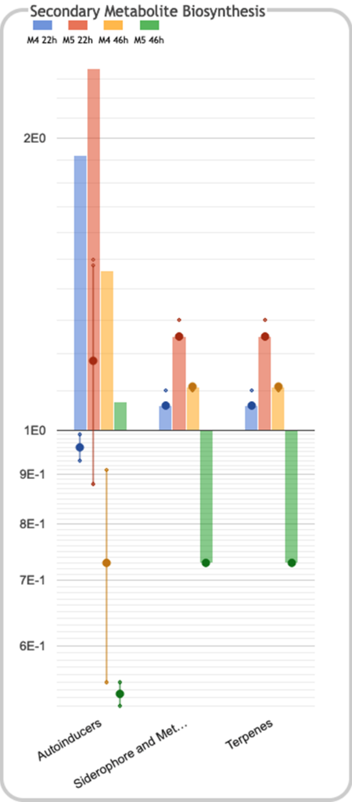
Secondary Metabolite Biosynthesis
M4 22h
M5 22h
M4 46h
M5 46h
2E0
1E0
9E-1
8E-1
7E-1
6E-1
Autoinducers
Siderophore and Met...
Terpenes

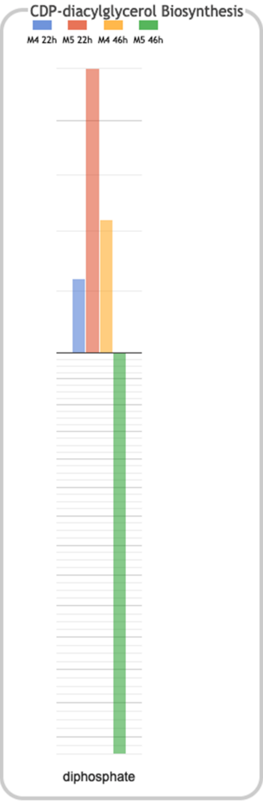
CDP-diacylglycerol Biosynthesis
M4 22h
M5 22h
M4 46h
M5 46h
diphosphate

b) Degradation

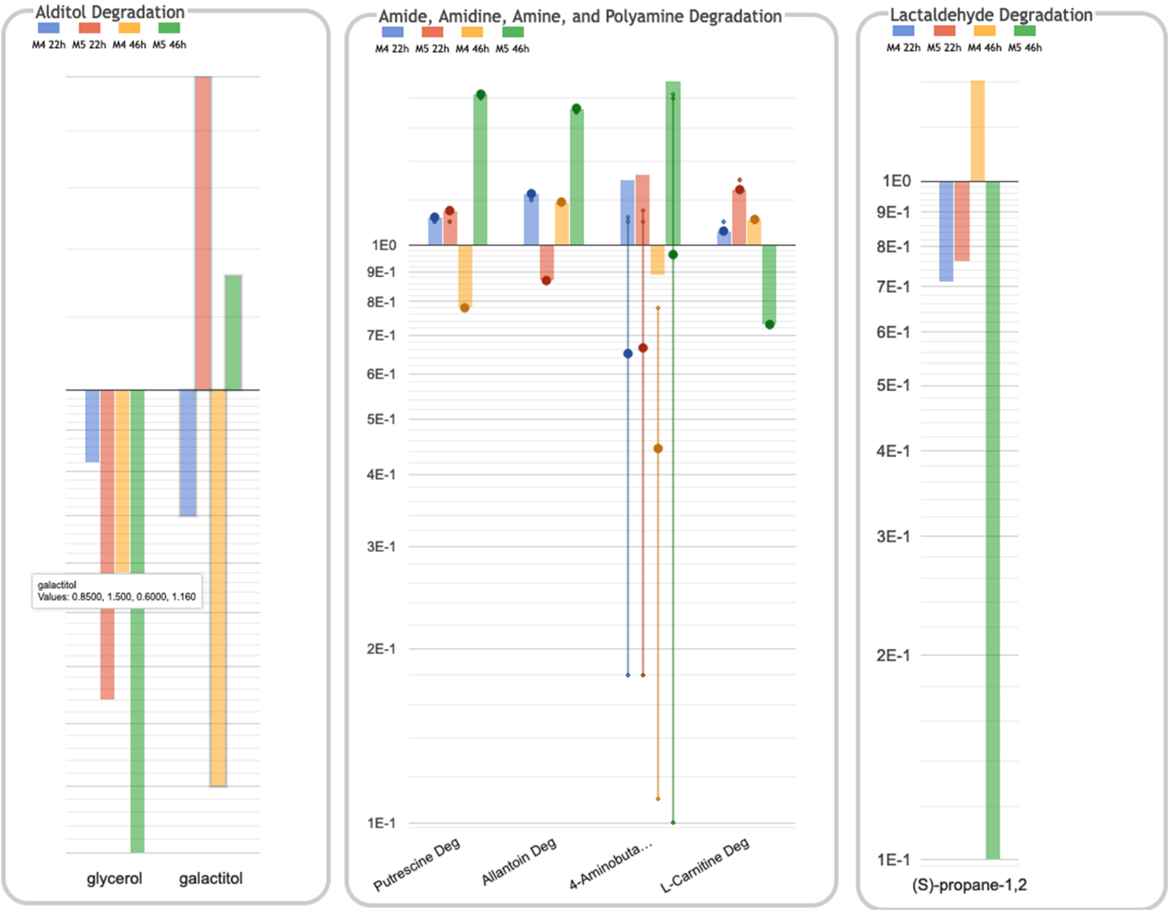

Figure S5. Box plot of intracellular (a) fumarate and (b) succinate peak's areas obtained in M4, M5 and wild type strains biomass collected at 22 and 46 h. In asterisk are shown statistical significant differences respect to wild type strain values with a *p-value* <0.001 (***).

a)

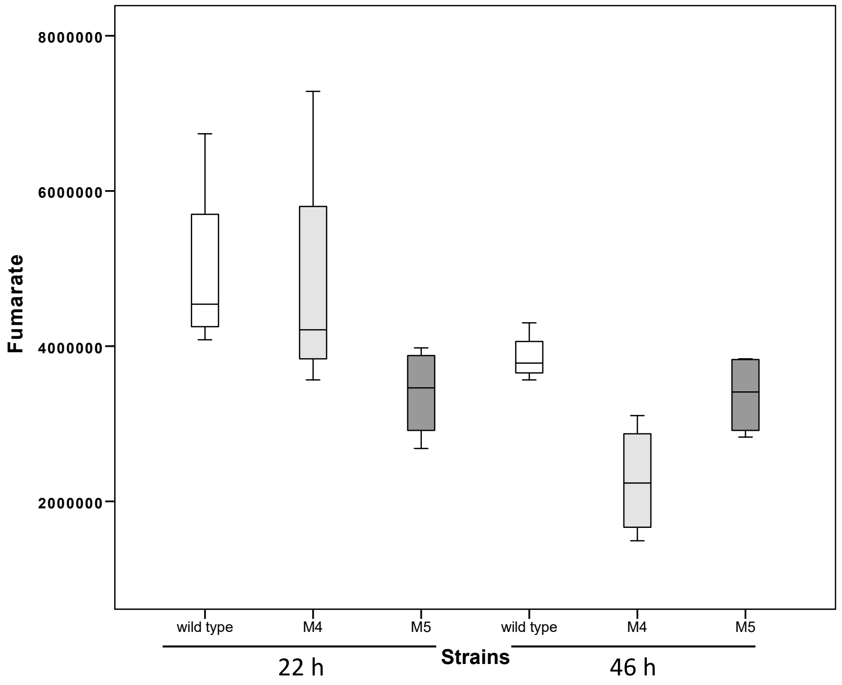

b)

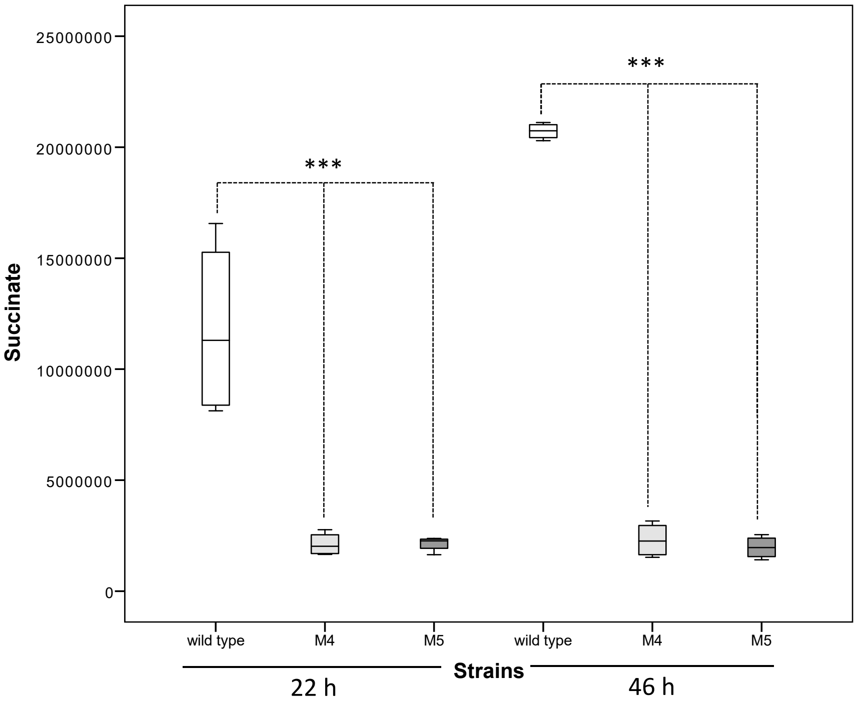

Figure S6. Scatter plots of average and standard deviations of: a) specific H_2 production; b) specific ethanol production; c) glycerol remained in mmol /gCDW; d) biomass growth in cell dried weight per liter (CDW/L), e) H_2 molar yield and f) ethanol molar yield in mmol/mmol glycerol consumed. These parameters have been obtained at 46 and 70 h in the following strains: M5/pB-K, M5/pB-K+pT (denoted as reference strains) M5/pB-K-CIT, M5/pB-K-CIT+pT-pepck, M5/pB-K-CIT+pBA-maeA (denoted as strains with overexpressions).

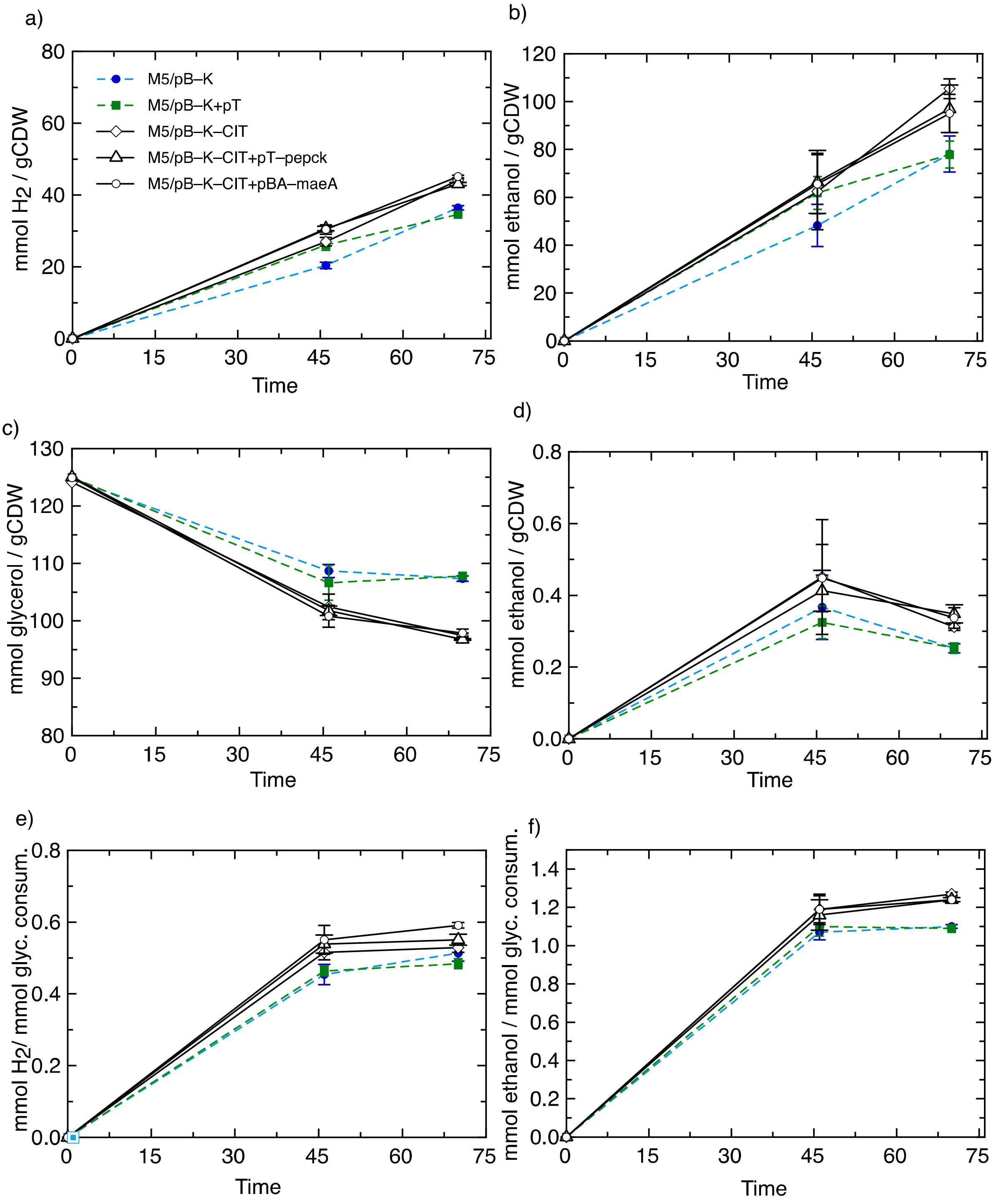

Supplement: Supplementary file 1 [file ijms-24-11619-s001.zip › ijms-2424186-supplementary.pdf]
